# Baf-mediated transcriptional regulation of teashirt is essential for the development of neural progenitor cell lineages

**DOI:** 10.1038/s12276-024-01169-3

**Published:** 2024-02-19

**Authors:** Byung Su Ko, Myeong Hoon Han, Min Jee Kwon, Dong Gon Cha, Yuri Ji, Eun Seo Park, Min Jae Jeon, Somi Kim, Kyeongho Lee, Yoon Ha Choi, Jusung Lee, Monica Torras-Llort, Ki-Jun Yoon, Hyosang Lee, Jong Kyoung Kim, Sung Bae Lee

**Affiliations:** 1 Department of Brain Sciences, DGIST, Daegu, 42988 Republic of Korea; 2 Department of New Biology, DGIST, Daegu, 42988 Republic of Korea; 3 Department of Life Sciences, Pohang University of Science and Technology (POSTECH), Pohang, 37673 Republic of Korea; 4 Convergence Research Advanced Centre for Olfaction, DGIST, Daegu, 42988 Republic of Korea; 5 Institute of Molecular Biology of Barcelona, CSIC, Barcelona, Spain; 6 Department of Biological Sciences, Korea Advanced Institute of Science and Technology (KAIST), 291 Daehak-ro, Yuseong-gu, Daejeon, 34141 Republic of Korea

**Keywords:** Computational biology and bioinformatics, Genetics

## Abstract

Accumulating evidence hints heterochromatin anchoring to the inner nuclear membrane as an upstream regulatory process of gene expression. Given that the formation of neural progenitor cell lineages and the subsequent maintenance of postmitotic neuronal cell identity critically rely on transcriptional regulation, it seems possible that the development of neuronal cells is influenced by cell type-specific and/or context-dependent programmed regulation of heterochromatin anchoring. Here, we explored this possibility by genetically disrupting the evolutionarily conserved *barrier-to-autointegration factor* (*Baf*) in the *Drosophila* nervous system. Through single-cell RNA sequencing, we demonstrated that *Baf* knockdown induces prominent transcriptomic changes, particularly in type I neuroblasts. Among the differentially expressed genes, our genetic analyses identified teashirt (tsh), a transcription factor that interacts with beta-catenin, to be closely associated with *Baf* knockdown-induced phenotypes that were suppressed by the overexpression of *tsh* or *beta*-*catenin*. We also found that Baf and tsh colocalized in a region adjacent to heterochromatin in type I NBs. Notably, the subnuclear localization pattern remained unchanged when one of these two proteins was knocked down, indicating that both proteins contribute to the anchoring of heterochromatin to the inner nuclear membrane. Overall, this study reveals that the Baf-mediated transcriptional regulation of *teashirt* is a novel molecular mechanism that regulates the development of neural progenitor cell lineages.

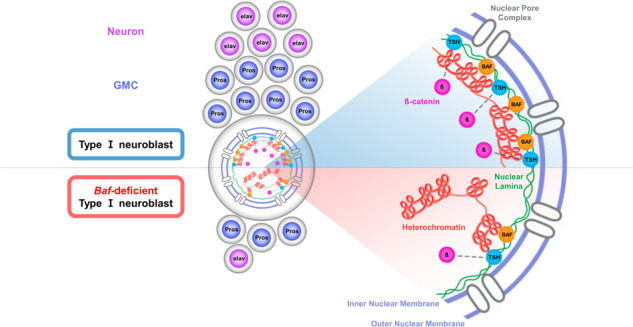

## Transcriptomic changes in neuroblasts: the impact of Baf knockdown

The development of nervous system requires a tightly controlled process of cell differentiation that originates from neural progenitor cells. Here, Ko and colleagues investigated the cellular and molecular basis underlying heterochromatin anchoring-dependent regulation of neural progenitor cell lineages by combining single-cell RNA sequencing and genetic analyses using *Drosophila*. They demonstrated the neurodevelopmental role of Barrier-to-autointegration factor (Baf), a chromatin-binding proteins mediating subnuclear positioning of heterochromatin to inner nuclear membrane, in a cell type-specific manner. Moreover, Ko and colleagues newly identified that cell type-specific transcriptional regulation of *teashirt* by Baf is essential for the formation of neural progenitor cell lineages. These findings deepen our understanding of the subnuclear positioning of heteorchromatin involved in the formation of neural progenitor cell lineages and subsequent neurodevelopment. This summary was initially drafted using artificial intelligence, then revised and fact-checked by the author.

## Introduction

The nervous system consists of diverse cellular components including various types of neurons and glial cells. The complex cellular composition of the nervous system requires the tightly controlled generation of specific cell types from neural progenitor cells during development, which involves asymmetric/symmetric cell division and spatiotemporal expression of transcription factors (TFs) that determine cell identity. Extensive studies have been conducted to identify factors regulating neurodevelopment and revealed that the orchestrated action of TFs is crucial for both the formation of neural progenitor cell lineages and the subsequent maintenance of postmitotic neuronal cell identity^1–3^. For instance, neural progenitors determine the cellular identity of their progenies^4,5^ through the sequential upregulation of temporal TFs, such as Hunchback (Hb), Kruppel (Kr), POU domain protein (Pdm), and Castor (Cas), which was first characterized in embryonic neuroblasts (NBs) of the *Drosophila* ventral nerve cord^6,7^. In addition, regarding the maintenance of postmitotic neuronal cell identity, the initiation and subsequent maintenance of olfactory receptor expression patterns in *Drosophila* olfactory sensory neurons are regulated by combinations of seven TFs (acj6, E93, Fer1, onecut, sim, xbp1, and zf30c)^8^. However, the upstream activators/cues or regulatory processes that control the activities and/or amounts of these TFs in cell type-specific and/or context-dependent (e.g., developmental stages or cell cycle phases) manners during nervous system development remain largely unknown.

In this regard, it is worth noting that recent studies have highlighted the roles of chromosomal features (i.e., higher-order chromatin organization and subnuclear positioning of chromatin) in the regulation of gene expression^9,10^. As for the nervous system, the roles of chromatin organization in neurodevelopment, specifically via transcriptional regulation, have recently been actively explored^11–14^, whereas the roles of subnuclear positioning of chromatin involving transcriptional regulation have remained relatively less understood. The anchoring of heterochromatin to the inner nuclear membrane (INM) is crucial for subnuclear positioning of chromatin^15,16^, which is mediated by chromatin-binding proteins/chromatin-anchoring proteins, such as the evolutionally conserved barrier-to-autointegration factor (Baf)^17–19^, mammalian proline-rich-protein (PRR14)^20^, and *C. elegans*-specific chromodomain-containing protein (cec-4)^21,22^. These chromatin-binding proteins/chromatin-anchoring proteins interact with lamin-associated proteins, such as proteins with LEM (LAP2, Emerin, MAN1) domains that reside in the INM^23–25^, to form physical interactions between the chromosome and the nuclear lamina. According to this anchoring function, investigations on the anchoring of heterochromatin to the INM have primarily focused on its involvement in chromosomal segregation and subsequent postmitotic reassembly of the nuclear envelope during mitosis^26–28^.

Beyond their well-characterized roles in mitosis, chromatin-binding proteins/chromatin-anchoring proteins can play a role in regulating gene expression. For instance, Baf is associated with the transcriptional regulation of target genes, which involves its direct interactions with promoter regions, recruitment of epigenetic modulators, and/or histone modifications^17,18,29^. Furthermore, accumulating evidence suggests the potential importance of heterochromatin anchoring to the INM in the differentiation of cells into specific cell types during development. A previous study reported that *baf-1*, a *C. elegans* ortholog of *Baf*, plays a postmitotic role in muscle and epidermal cells; homozygous *baf-1* mutants are able to grow into L4 larvae/young adults but exhibit cell type-specific defects, such as defects in muscle cell integrity and premature fusion of epidermal cells, accompanied by a reduced body size^29^. Moreover, PRR14 is associated with myoblast differentiation that involves transcriptional regulation of specific target genes (e.g., *MyoD*) through its interaction with HP1alpha^30^. In this respect, we here investigated whether heterochromatin anchoring-dependent regulation of transcription affects neurodevelopment in a cell type-specific and/or context-dependent manner using *Drosophila* as a model. To this end, we specifically focused on the evolutionally conserved Baf^31^, since there is no known fly homolog of PRR14 or cec-4. To genetically disrupt the programmed regulation of heterochromatin anchoring during nervous system development, we used *Baf*-knockdown (KD) flies instead of *Baf-*null mutant flies because complete knockout of *Baf* results in mortality during the larval–pupal transition^32^. By combining single-cell transcriptomic analysis with a series of genetic analyses, we found that Baf-mediated transcriptional regulation of a specific downstream molecule, teashirt (tsh), which interacts with beta-catenin in the Wnt signaling pathway, is involved in the Baf-dependent regulation of heterochromatin anchoring to the INM, primarily in type I NBs, and the subsequent formation of neural progenitor cell lineages in *Drosophila*.

## Materials and methods

### Fly stocks

The following fly stocks were obtained from the Bloomington *Drosophila* Stock Center (Indiana, USA): *elav*-Gal4 (BL8760), *nSyb*-Gal4 (BL51941), *repo*-Gal4 (BL7415), UAS-*Baf* RNAi (BL36108), UAS-*Baf* RNAi (KK102013), *OK371*-QF2 (BL66473), QUAS-*mCD8GFP* (BL30003), UAS-*Redstinger* (BL8546), *wor*-Gal4,UAS-*mira*.*GFP*/*CyO*, *ActGFP*;UAS-*His*-*RFP* (BL56555), *neoFRT40A* (BL8212), *l(2)k10210* (BL10980), UAS-*tsh* RNAi (BL35030), UAS-*msk* RNAi (BL33626), UAS-*mts* RNAi (BL38337), UAS-*CG5033* RNAi (BL42930), UAS-*Spindly* RNAi (BL34933), UAS-*Uba2* RNAi (BL63986), UAS-*mars* RNAi (BL33929), UAS-*U4*-*U6*-*60K* RNAi (BL55210), UAS-*Caf1*-*55* RNAi (BL34069), UAS-*blw* RNAi (BL28059), UAS-*vvl* RNAi (BL50657), UAS-*feo* RNAi (BL28926), UAS-*Hsp70Bc* RNAi (BL42626), UAS-*Rap1* RNAi (BL35047), UAS-*puf* RNAi (BL62477), UAS-*foxo* RNAi (BL32993), UAS-*ZnT49B* RNAi (BL31933), UAS-*Ranbp9* RNAi (BL33004), UAS-*CG43340* RNAi (BL41926), UAS-*tsh* (BL52216), UAS-*tsh* (BL52217), UAS-*msk* (BL23944), UAS-*Arm* (BL8369), UAS-*Arm*.*S10* (BL4782), and UAS-*Arm* RNAi (BL35004). UAS-*OdsH*-3xHA (F000261), UAS-*FoxK-*3xHA (F000615), and UAS-*msk*-3xHA (F004312) were obtained from FlyORF (Zurich, Switzerland). Type I NB-Gal4 (*ase*-Gal4) and type II NB-Gal4 (*wor*-Gal4, *ase*-Gal80) were kindly provided by J.A. Knoblich (IMBA, Austria). *Tub*-Gal80^TS^, UAS-*mCD8GFP*, and UAS-*mCD4GFP* were gifts from Y.N. Jan (University of California, San Francisco, CA). *elav*-Gal4, UAS-*mCD8GFP*, *hsflp*;*FRT40A*, and *Tub*-Gal80 were kindly provided by J. Chung (Seoul National University, Korea). All flies were raised at room temperature (25 °C) with 60% humidity.

Between two available transgenic lines for overexpression of *tsh*, we observed that overexpression of *tsh* in type I NBs using the BL52216 transgenic fly line, but not the BL52217 transgenic fly line, induces early lethality during the first instar larval stage. Thus, the genetic interaction between *Baf* and *tsh* was first examined in the BL52217 line and subsequently confirmed in the BL52216 line co-expressed with Gal80 in the parents to prevent the early death of the progeny through the maternal supply of Gal80, or vice versa.

### Culture of primary NBs for live imaging

Culture of primary NBs was performed as previously described^33^. Third-instar larvae were briefly washed with PBS. Larval brains were dissected in Dissection medium [Schneider’s medium (Sigma) supplemented with 10% fetal bovine serum and 2% Pen/Strep] and collected in cold Rinaldini solution (800 mg NaCl, 20 mg KCl, 5 mg NaH_2_PO_4_, 100 mg NaHCO_3_, and 100 mg glucose in 100 ml distilled water). After brief washing with Rinaldini solution, the brains were incubated in Dissociation medium [Rinaldini solution containing 0.5 mg/ml collagenase I and 1 mg/ml papain (Sigma)] for 1 h at 30 °C to dissociate the tissues into individual cells. After 1 h, the dissociated cells were washed with Rinaldini solution and then with Dissection medium. The dissociated cells were subsequently resuspended in 200 μl of Dissection medium and plated in poly-L-lysin-coated cell culture dishes (FD35-100, FluoroDish). The culture dishes were incubated in a 25 °C incubator for 1 h, after which 3 ml of primary cell culture medium [Schneider’s medium (Sigma) supplemented with 10% fetal bovine serum, 2% Pen/Strep, 20 mM L-glutamine, 5 μg/ml L-glutathione, 20 μg/ml insulin, and 5 μg/ml ecdysone] was added before imaging.

### Culture of primary NBs for immunocytochemistry

Culture of primary NBs was conducted as previously described^34^. Third-instar larvae were rinsed with 1x PBS. All the solutions that were used for primary culture were kept cold on ice. Larval brains were dissected in Dissection medium [90% L-glutamine-supplemented Schneider’s medium, 10% heat-inactivated FBS, 0.1% penicillin/streptomycin]. Using type I NBs localized in the central lobe of the brain, we removed ventral nerve cords from each dissected larval brain and collected a total of 5 pairs of third-instar larval brain lobes in ice-cold Rinaldini solution [800 mg NaCl, 20 mg KCl, 5 mg NaH_2_PO_4_H_2_O, 100 mg NaHCO_3_, 100 mg glucose, in 100 ml distilled water]. After brief washing with Rinaldini solution, the larval brain lobes were incubated in Dissociation medium [Rinaldini solution containing 0.5 mg/ml collagenase I] for 1 h at room temperature, followed by washing 3 times with Rinaldini solution. Then, 10 µl of Dissection medium was added to each of the larval brain samples. Subsequently, the brain lobes were homogenized by gentle trituration using a micropipette. Dissociated cells were seeded on the 0.01% poly-L-lysine-coated coverslip and incubated in a wet chamber for 1 h in room temperature to allow the dissociated cells to settle. To maintain the humidity inside the wet chamber, we used a microcentrifuge tube storage box and placed a small bucket of water on the bottom of the box. The cells were fixed with 3.7% formaldehyde for 15 min at room temperature and briefly washed three times every 2 min using 0.3% PBT (0.3% Triton X-100 in phosphate-buffered saline (PBS)). Then, the samples were blocked with 10% NDS containing 0.3% PBT for 1 h at room temperature and then incubated with primary antibodies diluted in 5% NDS containing 0.3% PBT overnight at 4 °C. After overnight incubation, the samples were washed three times every 10 min with 0.3% PBT containing Hoechst (1:200 dilution). The coverslips were mounted in ProLong^TM^ Diamond Antifade Mountant for confocal imaging.

### Immunohistochemistry and immunocytochemistry

For immunohistochemistry, larval or adult brains were dissected in 1x PBS and stained as previously described^35^. The following primary antibodies were used: rat anti-Dpn (ab195173, Abcam; 1:100 dilution); mouse anti-BrdU (347580, BD Biosciences; 1:250 dilution); rabbit anti-GFP (ab183734, Abcam; 1:100 dilution); mouse anti-GFP (ab11120, Abcam; 1:200 dilution); mouse anti-Pros (MR1A, Developmental Studies Hybridoma Bank; 1:10 dilution); rat anti-Miranda (ab197788, Abcam; 1:100 dilution); rabbit anti-PH3 (9701, Cell Signaling Technology, 1:100); rabbit anti-TH (AB152, MERCK; 1:200 dilution); mouse anti-ChAT (ChAT4B1, Developmental Studies Hybridoma Bank; 1:10 dilution); rabbit anti-5-HT (20080, IMMUNOSTAR; 1:500 dilution); and rabbit anti-GABA (A2052, Sigma-Aldrich; 1:100 dilution).

To detect these primary antibodies, the following secondary antibodies were used: Alexa 488-conjugated goat anti-rabbit (Thermo Fisher Scientific; 1:600 dilution), Alexa 555-conjugated goat anti-rat (Thermo Fisher Scientific; 1:600 dilution), Cy3-conjugated donkey anti-mouse (Jackson ImmunoResearch Laboratories; 1:200 dilution), and Alexa 647-conjugated goat anti-rat (Jackson ImmunoResearch Laboratories; 1:600 dilution). Images of immunostained brain samples were captured using Zeiss confocal microscopes.

For BrdU labeling of NBs, dissected larval brains were incubated in 40 ng/ml BrdU in Schneider medium for 30 min at room temperature and subsequently transferred to Schneider medium and incubated for 24 h at 4 °C. After incubation, the larval brains were washed three times with 0.3% PBT and fixed with 3.7% formaldehyde in 0.3% PBT for 30 min at room temperature. Next, the larval brains were exposed to 1 N HCl for 30 min and washed three times with 0.3% PBT. Then, the samples were stained as previously described^35^.

For Hoechst staining, dissected larval brains were incubated in 0.3% PBT containing 5 μg/ml Hoechst for 40 min at room temperature. After brief washing with 0.3% PBT, the larval brains were mounted in PBS prior to imaging.

The following primary antibodies were used for immunocytochemistry: rat anti-Dpn (ab195173; Abcam; 1:50 dilution), rabbit anti-H3K9me3 (ab8898; Abcam; 1:100 dilution), mouse anti-Arm (N2 7A1; Developmental Studies Hybridoma Bank; 1:100 dilution), and mouse anti-GFP (ab11120; Abcam; 1:100 dilution). Rabbit anti-Baf was kindly provided by Fernando Azorin (Institute of Molecular Biology of Barcelona, CSIC, Barcelona, Spain). Guinea pig anti-tsh antibody was a gift from Richard S. Mann (Department of Biochemistry and Molecular Biophysics, Columbia University, New York, NY). To detect these primary antibodies, the following secondary antibodies were used: Alexa 555-conjugated goat anti-rat (Thermo Fisher Scientific; 1:400 dilution), Alexa 647-conjguated goat anti-rabbit (Thermo Fisher Scientific; 1:400 dilution), Alexa 488-conjugated goat anti-mouse (Thermo Fisher Scientific; 1:400 dilution), Alexa 647-conjugated goat anti-guinea pig (Jackson ImmunoResearch Laboratories; 1:400 dilution), and Hoechst 34580 (Thermo Fisher Scientific; 1:200 dilution).

### RT‒PCR

RT‒PCR analysis was performed as previously described^36^. Total RNA was extracted from adult fly heads using an Easy-Blue system (iNtRON Biotechnology). cDNAs were synthesized from 3 µg of total RNA using GoScript Reverse Transcription (A2791; Promega) following the manufacturer’s standard protocol. For RT‒PCR analysis, each target gene was amplified with the corresponding primer set (Supplementary Table 1) using GoTaq G2 Master Mix (M7823; Promega) in a C1000 Thermal Cycler, C1000 Touch Thermal Cycler, or T100 Thermal Cycler system (Bio-Rad).

### Live imaging of cultured primary NBs

To monitor the cell division of NBs, live imaging of cultured primary NBs was performed using an LSM7 live confocal microscope (equipped with an imaging chamber, 25 °C) with a 40x water/1.2 NA objective. The cell division of the NBs was recorded for 3 h, and Z-stack images were acquired every 180 s at 1 μm intervals.

### Calculation of the circularity of NBs

The circularity of NBs was calculated as previously described^26^. Confocal images were processed to determine the maximum projection intensity, and the perimeter (P) and area (A) of the NBs were measured using ImageJ. The circularity of the NBs was subsequently calculated using the following formula: $P/(2\,\cdot\, \sqrt{\pi \,\cdot\, A})$.

### Sample preparation for single-cell RNA sequencing (scRNA-seq)

For single-cell sequencing, third-instar larvae were collected and prepared as previously described^33^. Third-instar larvae expressing denoted transgenes (*elav*-Gal4 and *elav*-Gal4/UAS-*Baf* RNAi) were dissected in 1x PBS and collected in cold 1x PBS. The collected larval brains were centrifuged at 800 × *g* for 5 min, after which the supernatants were replaced with dissociation buffer (0.6 mg/ml dispase, 0.15 mg/ml collagenase I, and 0.025% trypsin-EDTA). The larval brains were incubated in a thermoshaker (25 °C) for 15 min at 1000 rpm. The dissociated brain cells were washed with cold 1x PBS and resuspended in Dulbecco’s phosphate-buffered saline (DPBS) containing 0.04% bovine serum albumin. The dissociated brain cells were subsequently filtered through a 40-μm strainer (Flowmi® Cell Strainers) and collected for the subsequent steps related to single-cell sequencing.

### scRNA-seq

Third-instar larval brains were collected and prepared as previously described^33^. Briefly, third-instar larvae of the *elav*-Gal4 and *elav*-Gal4/UAS-*Baf* RNAi fly stocks were dissected in 1x PBS and collected in cold 1x PBS. The collected larval brains were centrifuged at 800 × *g* for 5 min, after which the supernatants were replaced with dissociation buffer (0.6 mg/ml dispase, 0.15 mg/ml collagenase I, and 0.025% trypsin-EDTA). The larval brains were incubated in a thermoshaker (25 °C) for 15 min at 1000 rpm. The dissociated brain cells were washed with cold 1x PBS and resuspended in Dulbecco’s phosphate-buffered saline (DPBS) containing 0.04% bovine serum albumin. These dissociated cells were filtered with a 40-μm strainer (Flowmi® Cell Strainers), fixed with cold 80% methanol and stored at −20 °C until all the replicates were collected. To rehydrate the samples, the cells were centrifuged at 3000 × *g* for 5 min at 4 °C, after which the supernatants were removed. The cell pellets were washed 3 times with 1% BSA and 1% Superase • In RNase inhibitor (Ambion) in DPBS. The cells were resuspended at a concentration of 1 × 10^6^ cells/ml in cold 0.5x DPBS supplemented with an RNase inhibitor (Enzynomics).

The sequencing libraries for scRNA-seq were generated by SPLiT-seq^37^. Briefly, for in situ reverse transcription of the first-round barcode, cells were aliquoted into 48 wells of a 96-well plate, in which barcoded well-specific reverse transcription primers had been previously aliquoted. The second- and third-round barcodes were appended to the cDNA by an in-cell ligation step. After the third-round barcodes were appended, 8000 cells were aliquoted into each sublibrary and lysed. cDNA was purified and amplified using PCR. The quality and quantity of cDNA were monitored using a Bioanalyzer high sensitivity kit (Agilent). A total of 600 pg of cDNA was used for tagmentation, and the i5/i7 sample index was inserted by PCR. Purified libraries were sequenced on the Illumina HiSeq X platform (paired-end 150-bp reads) aiming for a depth of 50,000 read pairs per cell.

### scRNA-seq data preprocessing

The raw fastq files were processed using the zUMIs pipeline (v2.5.4)^38^. To extract UMI and cell barcode (CB) information from paired-end reads, the following base definitions were used in the YAML file: cDNA (1–151) from read 1, CB (11–18, 49–56, and 87–94) from read 2, and UMI (1–10) from read 2. The cDNA sequences were subsequently mapped to the *Drosophila* genome (BDGP6.28) using the STAR (v2.7.4a) aligner^39^ with the BDGP6.28.99 GTF file. A gene-by-cell count matrix was generated with default parameters, and cells with less than 100 total UMI counts were removed because the expected number of cells across samples was approximately 8000 cells. To filter out low-quality cells, cells with a total log10-scaled UMI count less than 2.2 and cells with more than 5% of the UMIs assigned to mitochondrial genes were excluded, and the thresholds were determined by visually inspecting the outliers in the PCA plot on the quality control metrics using the calculateQCMetrics function of the scater (v1.14.0) R package^40^. To remove cell-specific biases, cells were clustered using the quickCluster function of the scran (v1.14.6) R package^41^ with default parameters, and cell-specific size factors were computed using the computeSumFactors function of the same package. The aggregated gene-by-cell count matrix across samples was normalized by dividing the raw UMI counts by cell-specific size factors. The normalized counts were then log2-transformed by adding a pseudocount of 1. We defined highly variable genes (HVGs) as the 1000 genes with the highest biological variability using the decomposeVar function of the scran package. All the cells were grouped into 14 clusters using the FindClusters function of the Seurat (v3.2.0) R package^42^ on the first 10 PCs of HVGs with a resolution = 0.8 and visualized with a two-dimensional UMAP plot using the RunUMAP function of the same package on the same 10 PCs. Two clusters (Clusters 7 and 12) expressing multiple cell type markers were considered putative doublets and removed. The remaining cells were regrouped into 13 clusters on the 20 PCs of 1500 HVGs using the same method described above. For the analysis of neuronal lineage cells, neuronal cells (glial cells were excluded) were reclustered and visualized using the 7 PCs of 1500 HVGs.

### scRNA-seq data analysis

We identified differentially expressed genes (DEGs) in each cell type between the control and *Baf* KD groups using the limma (v3.44.3) R package^43^ with an adjusted *P* < 0.05 and an absolute value of log_2_FC > 0.1. Cell types responsive to *Baf* KD were prioritized based on the cross-validation area under the receiver operating characteristic curve (AUC) of the random forest classifier, which was implemented in the Augur (v1.0.0) R package with default parameters^44^. To determine the biological processes in which DEGs in each cell type between the control and *Baf* KD groups were enriched, significantly enriched GO biological process (GOBP) terms (*P* < 0.05) were selected using the topGO (v2.40.0) R package with the org.Dm.eg.db (v3.11.4) annotation data package. We performed pseudotime analysis of both the type I NB lineage (type I NB – GMC – neuron) and the type II NB lineage (type II NB – INP– GMC – neuron) under control or *Baf* KD conditions using the Palantir (v0.2.6) python package^45^. A k-nearest neighbor (kNN) graph (k = 30) was constructed using the first 10 diffusion components (DCs) derived from the 100 PCs of 1500 HVGs and visualized in the t-SNE plot based on the same 10 DCs. The starting cells for the pseudotime analysis were defined by choosing the cell with the highest expression of *ase* for the type I NB lineage and *pnt* for the type II NB lineage. A lineage tree in the same UMAP plot was generated for each lineage under each condition after separating cells according to their lineage and treatment condition. We constructed a kNN graph using the pp.neighbors function of the scanpy (v1.5.1) python package with 7 PCs. The connectivity of the cell types in each lineage under each condition was quantified using the tl.paga function of the same package with default parameters. The edge weights were visualized using the pl.paga function of the same package with the root partitions and layout Reingold-Tilford. To compare the neuronal development of each NB lineage between the control and *Baf* KD groups, we aligned two trajectories using the cellAlign (v0.1.0) R package^46^ with the Palantir pseudotime values of cells and 300 interpolated points for each trajectory.

### Behavioral analyses of adult flies

After eclosion from pupae, adult male flies were collected in vials containing fresh food and reared (25 °C, 60% humidity, and 10 AM:10 PM light:dark cycle) for 3–4 days. Then, each fly was separately transferred to a single fresh vial without CO_2_ anesthesia 12 h before behavioral analyses. On the day of the analyses, each fly was individually transferred to a recording chamber without CO_2_ anesthesia for the behavioral analyses. After 3 min of acclimation, the behavior of each fly was recorded for 10 min.

### Quantification and statistical analysis

For statistical comparisons of the quantified results between two different groups, two-tailed Student’s t-tests were used. When comparing three or more groups, one- or two-way ANOVA was used with Tukey’s post hoc correction. All these comparisons were performed using GraphPad Prism (version 7, GraphPad Software, Inc.). N.S., *, **, ***, and **** indicate *P* > 0.05, *P* < 0.05, *P* < 1.0 × 10^−2^, *P* < 1.0 × 10^−3^, and *P* < 1.0 × 10^−4^, respectively. All the data are shown as the mean ± SEM. The value of n represents the number of animals.

## Results

### scRNA-seq revealed that *Baf* KD induces prominent transcriptional changes, particularly in type I NBs

**Fig. 1:**
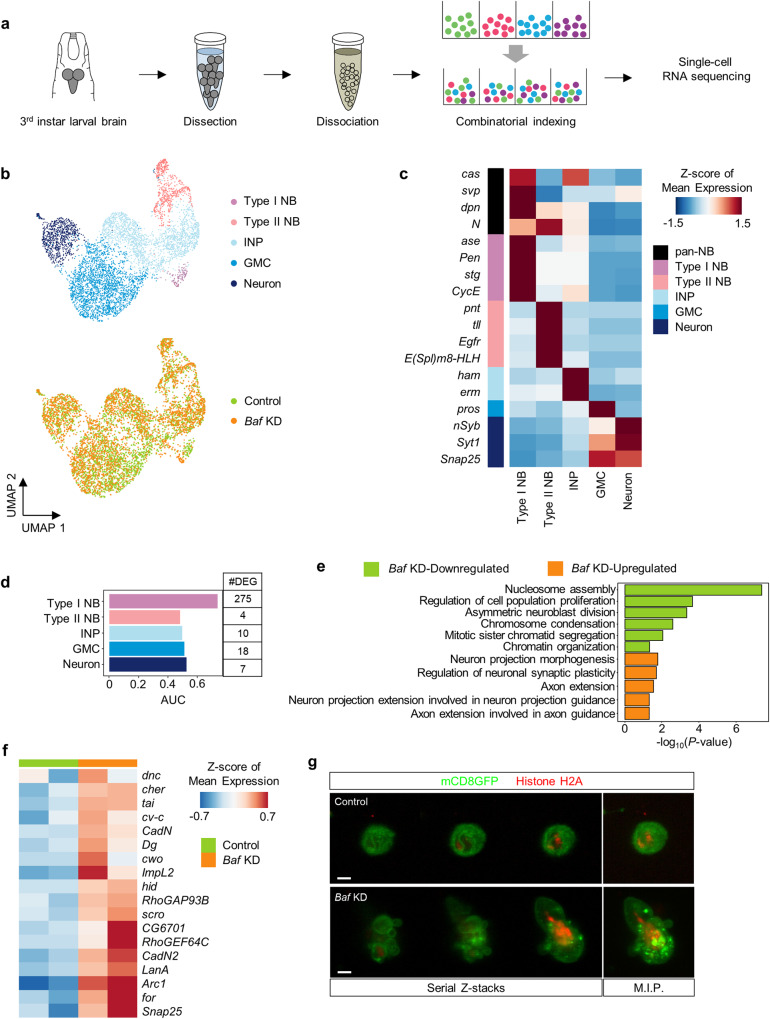
Single-cell characterization of the disrupted transcriptional profiles in type I NBs induced by *Baf* KD. **a** A schematic diagram of the combinatorial indexing-based scRNA-seq pipeline for third-instar larval brains. **b** UMAP plots showing all cells individually colored according to their cell type (*top*) or treatment condition (*bottom*). **c** Heatmap displaying the Z scores of the mean normalized expression of cell type-specific markers in each cell type. **d** Bar plot showing the AUC of each cell type indicating the separability of the control and *Baf* KD cells according to their transcriptomes. The number of each cell type and DEGs are shown in the additional table (*right*). **e** GO enrichment analysis of upregulated (orange) and downregulated (green) genes in type I NBs lacking *Baf*. **f** Heatmap showing the Z scores of the mean normalized expression of neurite-related genes that were upregulated in type I NBs lacking *Baf*. **g** Representative images of cultured primary type I NBs that were isolated from control larval brains (*top*, *ase*-Gal4/+;UAS-*mCD8GFP*,UAS-*H2A.mRFP*/+) or from larval brains expressing *Baf* RNAi (*bottom*, *ase*-Gal4/+;UAS-*mCD8GFP*,UAS-*H2A.mRFP*/UAS-*Baf* RNAi). Cultured primary type I NBs were visualized by the expression of the fluorescent plasma membrane marker protein, mCD8GFP. The *left* three panels show serial z-stack images, and the *right* panels show images processed with maximum intensity projection (M.I.P.) (Scale bar, 5 μm).

To understand the neurodevelopmental roles of Baf, we first characterized the cell type-specific transcriptional changes induced by *Baf* deficiency by performing combinational indexing-based single-cell RNA sequencing (scRNA-seq) of all brain cells (Fig. 1a). To this end, we collected third-instar larval brains from control larvae and larvae expressing *Baf* RNAi driven by *elav*-Gal4. Between two available *Baf* RNAi transgenes (BL36108 and KK102013), we used only the BL36108 line for scRNA-seq due to the off-target identified in the KK102013 line. Additionally, we confirmed the efficiency of the *Baf* RNAi (BL36108) transgene (Supplementary Fig. 1a, b). Thus, hereafter, “*Baf* RNAi” refers to the BL36108 transgenic line unless otherwise specified. From two pooled replicates for each condition, a total of 6365 cells (3176 control larval brain cells and 3189 *Baf* KD larval brain cells) passed our quality control criteria (Supplementary Fig. 1c–e; Supplementary Table 2). We applied a graph-based unsupervised clustering algorithm to systematically characterize the distinct cell types in the larval brain, resulting in 14 distinct clusters that were organized into 7 cell types (Supplementary Fig. 1c). After excluding glial cells, we subclustered 5949 neuronal cells into 13 distinct clusters that were organized into 5 cell types based on the expression of known cell type-specific markers (Fig. 1b, c and Supplementary Fig. 1f). Since all the samples were processed in the same batch, our scRNA-seq data showed minimal batch effects (Supplementary Fig. 1g–i). The two NB subsets (type I and II NBs) expressing pan-NB markers (*dpn* and *N*)^47,48^ were distinguished by the expression of *ase* (type I NB)^49^ and *pnt* (type II NB)^50^. The ganglion mother cell (GMC) population was defined by the expression of a canonical GMC marker (*pros*)^51,52^ and the absence of NB marker expression. The intermediate neural progenitor (INP) markers (*ham* and *erm*)^53^ were highly expressed in the INP population. The neuronal population was identified by the expression of neuronal markers (*nSyb*, *Syt1*, and *Snap25*). Additional cell type-specific marker genes that were recently used to annotate cell types in previous scRNA-seq studies^54,55^ were also considered in the grouping of cells into 5 different types (Fig. 1c).

To determine which cell types in larval brains were most affected by *Baf* deficiency, we first quantified the transcriptomic differences in each cell type between the two conditions with respect to the number of differentially expressed genes (DEGs) and the separability of *Baf* KD and control cells according to their transcriptomes. Based on these two measures, type I NBs exhibited the most prominent changes in response to *Baf* deficiency (Fig. 1d and Supplementary Fig. 1j). Functional enrichment analysis of the DEGs in type I NBs revealed enrichment of biological processes that are crucial for the functionality and morphogenesis of differentiated neurons among the upregulated genes in the *Baf* KD group and enrichment of biological processes that are essential for cellular division among the downregulated genes in the *Baf* KD group (Fig. 1e). These data suggest that *Baf* KD specifically induces an aberrant shift in the transcriptional profiles of type I NBs toward those of differentiated neurons, thereby resulting in impaired formation of neural progenitor cell lineages. Notably, the biological processes that were enriched in the upregulated genes in the *Baf* KD group included genes that are involved in the regulation of neuronal morphology, such as *ImpL2*, *RhoGAP93B*, *Snap25*, *for*, *CadN2*, *LanA*, and *scro* (Fig. 1f). This finding is consistent with the observations that, compared with cultured control type I NBs, cultured primary type I NBs isolated from third-instar larval brains expressing *Baf* RNAi often exhibited bulging of the plasma membrane, failure to maintain a circular morphology, and increased diameter (Fig. 1g and Supplementary Fig. 1k, l). Taken together, these results demonstrate that *Baf* KD induces prominent transcriptional changes, particularly in type I NBs.

### *Baf* KD in type I NBs causes defective anchoring of heterochromatin to the INM and impairs the formation of intact NBs and their neural progenies

**Fig. 2:**
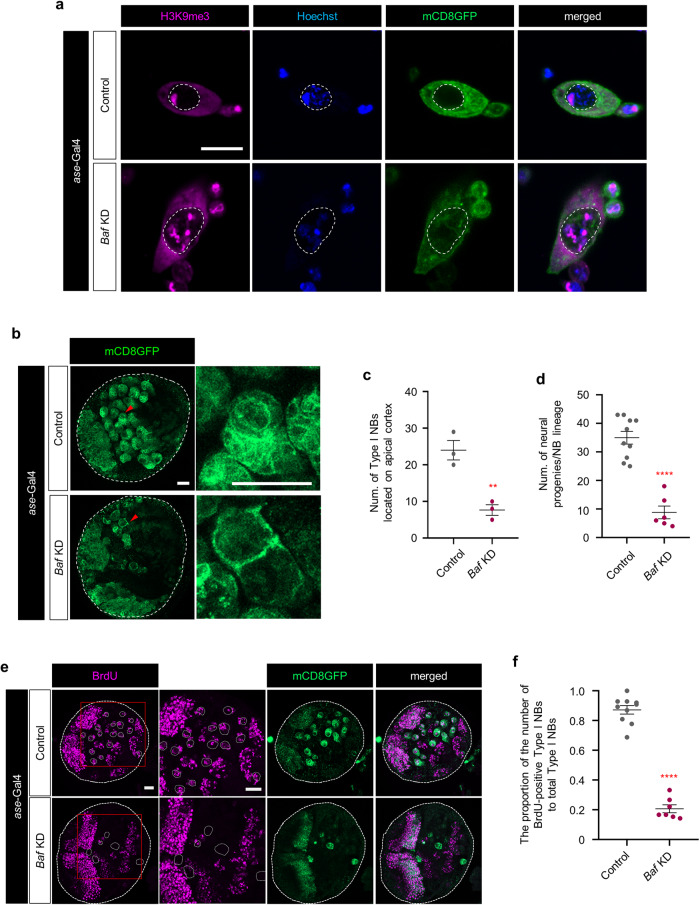
Aberrant anchoring of heterochromatin to the INM and perturbed formation of type I NBs and their neural progenies induced by *Baf* deficiency. **a** Representative images of immunostaining for H3K9me3 with Hoechst staining in cultured primary control type I NBs and type I NBs expressing *Baf* RNAi that were isolated from the brains of third-instar larvae (*ase*-Gal4/+;UAS-*mCD8GFP*/+ and *ase*-Gal4/+;UAS-*mCD8GFP*/UAS-*Baf* RNAi). NBs are visualized by the expression of the fluorescent plasma membrane marker protein mCD8GFP. The white dashed lines in the panels indicate the outlines of the inner nuclear membrane (Scale bar, 10 μm). **b** Representative images of control type I NBs (top, *ase*-Gal4/+;UAS-*mCD8GFP*/+) or type I NBs expressing *Baf* RNAi (bottom, *ase*-Gal4/+;UAS-*mCD8GFP*/UAS-*Baf* RNAi) in the brains of third-instar larvae. NBs are visualized by the expression of the fluorescent plasma membrane marker protein mCD8GFP. The white dashed lines in the left panels indicate the outlines of the brain lobes. Magnified images of type I NBs indicated by red arrowheads in the left panel are presented in the right panels (Scale bars, 20 μm). **c** Quantification of the number of type I NBs located in the apical cortex of third-instar larval brain lobes expressing the transgenes indicated in (**b**). **P < 1.0 × 10^−2^ according to Student’s t-test; error bars, mean ± SEM; the number of brain lobes tested was as follows: Control = 3 brain lobes, *Baf* KD = 3 brain lobes. **d** Quantification of the number of neural progenies derived from type I NBs located in the apical cortex of third-instar larval brain lobes expressing the transgenes indicated in (**b**). ****P < 1.0 × 10^−4^ according to Student’s t-test; error bars, mean ± SEM; the number of brain lobes tested was as follows: Control = 10 NBs, *Baf* KD = 6 NBs. **e** Representative images of BrdU (magenta) staining in control type I NBs and type I NBs expressing *Baf* RNAi in the brains of third-instar larvae. (*ase*-Gal4/+;UAS-*mCD8GFP*/+ and *ase*-Gal4/+;UAS-*mCD8GFP*/UAS-*Baf* RNAi). Magnified images of the red squares in the leftmost panels are presented in the second left panels. The white dashed lines in the panels indicate the outlines of the NBs (Scale bars, 20 μm). **f** Quantification of the proportion of BrdU-positive type I NBs to total type I NBs located in the apical cortex of third-instar larval brain lobes expressing the transgenes indicated in (**e**). ****P < 1.0 × 10^−4^ according to Student’s t-test; error bars, mean ± SEM; the number of brains tested is as follows: Control = 10 brain lobes, *Baf* KD = 7 brain lobes.

We next explored the cellular defects of type I NBs lacking *Baf*. We first examined whether the subnuclear distribution of heterochromatin is altered by *Baf* deficiency through immunostaining for H3K9me3, a representative marker of constitutive heterochromatin^56^, in cultured primary type I NBs. We observed that heterochromatin labeled by H3K9me3 in control type I NBs tended to localize near the nuclear periphery, whereas that in type I NBs lacking *Baf* was either abnormally localized and enriched in the center of the nucleus (data not shown) or discontinuously scattered throughout the entire genome (Fig. 2a), indicative of defective subnuclear positioning of chromatin in *Baf*-deficient type I NBs.

Then, we investigated the phenotypic changes of type I NBs in third-instar larval brains (Supplementary Fig. 2a) due to *Baf* deficiency. To this end, we knocked down *Baf* by *ase*-Gal4^49^-driven expression of the *Baf* RNAi transgene in type I NBs of *Drosophila*. The number of type I NBs and their neural progenies were significantly decreased in brains expressing *Baf* RNAi compared to control brains (Fig. 2b–d), which was similarly observed in brains expressing the other *Baf* RNAi (KK102013), having one off-target (data not shown). Importantly, more than half of the NBs expressing either *Baf* RNAi showed marked defects in the formation of NBs and their neural progenies (magnified images in Fig. 2b and data not shown), indicative of defects in the self-renewal of type I NBs and the subsequent formation of neural progenitor cells. On the other hand, since our scRNA-seq data showed that *Baf* KD induced prominent transcriptional changes, particularly in type I NBs, we examined whether *Baf* KD induces cellular defects specifically in type I NBs but not in type II NBs. For this purpose, we selectively knocked down *Baf* in type II NBs using *wor*-Gal4 together with *ase*-Gal80 to prevent Gal4 expression in type I NBs^57^. *Baf* KD in type II NBs had a very marginal but not statistically significant effect on the formation of type II NBs and their neural progenies (Supplementary Fig. 2b–d), indicating that type I NBs are particularly vulnerable to the reduced dosage of the *Baf* gene during development of the *Drosophila* nervous system.

According to previous studies, *Baf* deficiency leads to defects in general cell division in both cultured mammalian cells and *Drosophila*^26,58,59^, which is consistent with our findings of defective self-renewal in type I NBs lacking *Baf* (Fig. 2b, c). Consistent with these findings, live imaging of cultured primary type I NBs expressing *Baf* RNAi displayed defective cell division accompanied by ectopically increased histone H2A levels compared to those of cultured control type I NBs (Supplementary Fig. 2e). In addition to the elevated levels of histone H2A, we also observed increased DNA contents in type I NBs lacking *Baf* compared to those in the control group. (Supplementary Fig. 2f, g). Increased DNA contents are indicative of defective cell division resulting from defective chromosomal segregation during cell division^60^. Furthermore, the significant reduction in the number of cells that contained phosphorylated histone, a specific marker of both mitosis and meiosis^61^, among type I NBs in larval brains expressing *Baf* KD also supported the occurrence of defective cell division in type I NBs lacking *Baf* (Supplementary Fig. 2h, i). We thus inferred that the increased levels of histone H2A are accompanied by an increase in DNA content in type I NBs deficient for *Baf* to maintain chromosomal structure within the nucleus. To further support this, we examined another histone protein, histone H3, and found that the level of histone H3 was also increased in type I NBs lacking *Baf* (Supplementary Fig. 2j). Overall, we concluded that heightened levels of histone H2A could be considered a cellular feature of defective cell division, consistent with the increased DNA contents in type I NBs lacking *Baf*. To further confirm the defect in the division of type I NBs caused by *Baf* deficiency, we explored whether the proliferation of NBs is indeed impaired by *Baf* deficiency by performing BrdU labeling of NBs in larval brains lacking *Baf* and those of controls. The proportion of BrdU-positive type I NBs among the total type I NBs in larval brains expressing *Baf* RNAi was significantly decreased compared to the control (Fig. 2e, f), indicating the defective proliferation of type I NBs caused by *Baf* deficiency. Taken together, *Baf* KD in type I NBs causing defective anchoring of heterochromatin to the INM impairs the formation of intact NBs and their neural progenies along with defective cell division.

### Type I NB-specific neurodevelopment is substantially impaired by *Baf* KD, leading to a marked reduction in excitatory neurons in the adult stage

**Fig. 3:**
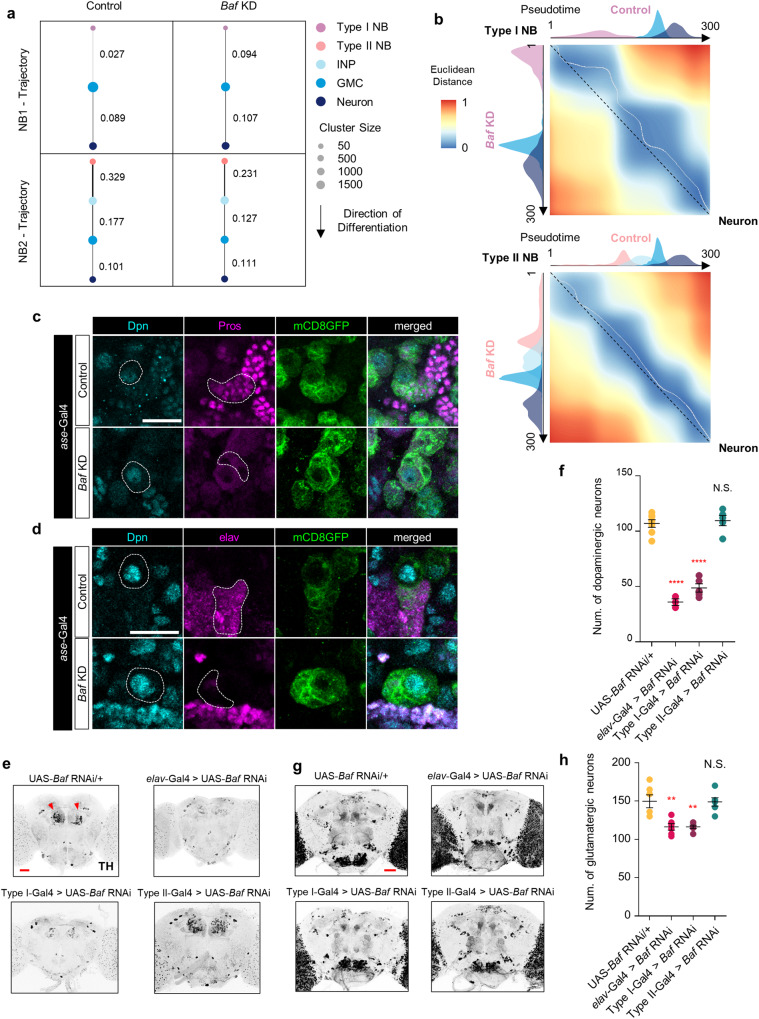
Impaired neurodevelopment of type I NB lineages induced by *Baf* KD. **a** PAGA graphs showing connectivity between cell types. The values on the right of each edge indicate the connectivity between cell types. **b** Dissimilarity matrices of global alignments of developmental trajectories of type I (top) and II (bottom) NB lineages. The histograms on each axis illustrate the individual cell types. **c** Representative images of immunostaining for Dpn (teal) and Pros (magenta) in control type I NBs and type I NBs expressing *Baf* RNAi in the brains of third-instar larvae (*ase*-Gal4/+;UAS-*mCD8GFP*/+ and *ase*-Gal4/+;UAS-*mCD8GFP*/UAS-*Baf* RNAi). The white dashed lines indicate the outlines of NBs (first column) and neural progenies (*second* column) (Scale bar, 20 μm). **d** Representative images of immunostaining for Dpn (teal) and elav (magenta) in type I NBs in the brains of third-instar larvae expressing the transgenes indicated in (**d**). The white dashed lines indicate the outlines of NBs (first column) and neural progeny cells (*second* column) (Scale bar, 20 μm). **e** Representative images of immunostaining for TH in adult brains of the following genotypes: UAS-*Baf* RNAi/+, *elav*-Gal4/UAS-*Baf* RNAi, *ase*-Gal4/+;UAS-*Baf* RNAi/+, and *wor*-Gal4,*ase*-Gal80/+;UAS-*Baf* RNAi/+. The red arrowheads indicate dopaminergic neurons (Scale bar, 40 μm). **f** Quantification of the number of dopaminergic neurons in the brains of flies expressing the indicated transgenes (UAS-*Baf* RNAi/+, *elav*-Gal4/+;UAS-*Baf* RNAi, *ase*-Gal4/+;UAS-*Baf* RNAi/+, and *wor*-Gal4,*ase*-Gal80/+;UAS-*Baf* RNAi/+). N.S., not significant; ****P < 1.0 × 10^−4^ by one-way ANOVA with Tukey’s post hoc test; error bars, mean ± SEM; UAS-*Baf* RNAi/+ = 7 brains, *elav*-Gal4 > *Baf* RNAi = 3 brains, type I-Gal4 > *Baf* RNAi = 5 brains, and type II-Gal4 > *Baf* RNAi = 5 brains. **g** Representative images showing the distribution and pattern of glutamatergic neurons in adult brains harboring the following genotypes: *OK371*-QF2/+;QUAS-, *mCD8GFP*/UAS-*Baf* RNAi, *OK371*-QF2/*elav*-Gal4;QUAS-*mCD8GFP*/UAS-*Baf* RNAi, *OK371*-QF2/*ase*-Gal4;QUAS-*mCD8GFP*/UAS-*Baf* RNAi, and *OK371*-QF2/*wor*-Gal4,*ase*-Gal80;QUAS-*mCD8GFP*/UAS-*Baf* RNAi. (Scale bar, 50 μm). **h** Quantification of the number of glutaminergic neurons in the brains of flies expressing the indicated transgenes (*OK371*-QF2/+;QUAS-*mCD8GFP*/UAS-*Baf* RNAi, *OK371*-QF2/*elav*-Gal4;QUAS-*mCD8GFP*/UAS-*Baf* RNAi, *OK371*-QF2/*ase*-Gal4;QUAS-*mCD8GFP*/UAS-*Baf* RNAi, and *OK371*-QF2/*wor*-Gal4,*ase*-Gal80;QUAS-*mCD8GFP*/UAS-*Baf* RNAi). N.S., not significant; **P < 1.0 × 10^−2^ by one-way ANOVA with Tukey’s post hoc test; error bars, mean ± SEM; *n* = 6 brains.

We next examined the developmental trajectories of type I and II NBs during neurogenesis in the brains of control larvae and larvae expressing *Baf* RNAi by using partition-based graph abstraction (PAGA)^62^. In the type I and II NB lineages, PAGA predicted a linear trajectory (type I NB – GMC – neuron or type II NB – INP – GMC – neuron) under both conditions (Fig. 3a). This prediction is consistent with the established temporal fate specification of NBs^63^. We confirmed the robustness of our predicted trajectories using Palantir^45^ (Supplementary Fig. 3a) and further analyzed the *Baf* KD-induced changes in expression dynamics along these developmental trajectories by aligning the two trajectories under both conditions. Similar to the analysis of regulator expression dynamics in the control group, the two trajectories of type II NBs were largely well aligned (Fig. 3b). However, we identified an off-diagonal alignment in early pseudotime for the trajectories of type I NBs (Fig. 3b). We then evaluated whether the perturbed linear trajectory of type I NBs compared to that of type II NBs is attributed to changes in the expression of related genes affected by *Baf* deficiency. To this end, we sorted the genes into 5 modules and conducted Gene Ontology (GO) analysis (Supplementary Fig. 3b, c). Within the linear trajectory of type I NBs, *Baf* deficiency disrupted the expression of a subset of transcriptional regulators that are associated with biological processes, including neuroblast fate determination (Dr^64^), neural precursor cell proliferation (SoxN^65^), regulation of cell cycle (cycA^66^, E2f1^67^), chromatin remodeling (HmgD^68^), and chromatin assembly or disassembly (polybromo^69^) in module M1 (Supplementary Fig. 3d). Furthermore, genes involved in the negative regulation of cell fate specification (*aop*^50,70^, *med4*^71^, and *med10*^71^) and negative regulation of cell proliferation (*hfp*^70^) were upregulated in the type I NB linear trajectory (module M2) (Supplementary Fig. 3d). Conversely, along the linear trajectory of type II NBs, *Baf* deficiency did not induce noticeable changes in the expression of the genes identified in modules M1 and M2 of the linear trajectory of type I NBs (Supplementary Fig. 3d). Taken together, these data indicate that *Baf* deficiency specifically impairs the fate specification of cells derived from type I NBs during neurodevelopment.

To experimentally validate the impaired fate specification of neural progenies derived from type I NBs caused by *Baf* deficiency, we first examined whether the formation of the polarity axis of NBs, which is important for their asymmetric division^72–74^ and the production of their neural progenies, is affected by *Baf* deficiency in type I NBs. According to previous studies, the asymmetric distribution of proteins that regulate self-renewal or differentiation of daughter cells is essential for the asymmetric cell division of NBs^75,76^. To achieve this, atypical protein kinase C (aPKC), a component of the par complex, localizes to the apical cortical region and restricts basal domain components, such as Miranda (Mira), to the basal region of NBs^72,73^. Mira, an adaptor protein of Pros and Brat, localizes to the basal cortex through direct aPKC phosphorylation, which contributes to the polarized distribution of fate determinants during asymmetric cell division of NBs^73,77^. Based on these findings, we examined the distribution and expression of aPKC and Mira in cultured primary type I NBs lacking *Baf* by immunostaining. While asymmetric and polarized distributions of both aPKC (apical distribution) and Mira (basal distribution) were detected in control Type I NBs, aPKC and Mira were almost undetectable in type I NBs deficient for *Baf* (Supplementary Fig. 3e). We could occasionally observe detectible signals of Mira in type I NBs lacking *Baf*, and in these cells, Mira exhibited a dispersed and unpolarized distribution (data not shown) instead of the asymmetric and polarized distribution that was observed in the control. The perturbed formation of the polarity axis in NBs suggested that asymmetric cell division in type I NBs is impaired by *Baf* deficiency, which contributes to the malformation of neural progenitor cell lineages. We then examined whether *Baf* deficiency in type I NBs leads to alterations in the expression of cell fate determinants in individual neural progenies derived from type I NBs. For this purpose, we labeled type I NBs and their neural progenies by immunostaining Dpn and cell fate determinants (Pros for GMC and elav for neuron), respectively, in control larval brains and larval brains lacking *Baf*. Notably, the neural progenies derived from type I NBs lacking *Baf* showed decreased level of each cell fate determinant compared to that of the control (Fig. 3c, d). These results suggest that *Baf* deficiency impairs the fate specification of neural progeny derived from type I NBs.

We then examined whether *Baf* deficiency in type I NBs during larval development leads to alterations in the cellular composition of the nervous system in adult flies. To this end, we first labeled representative excitatory neurons, such as dopaminergic (DA) and glutamatergic neurons, in control adult brains and adult brains expressing *Baf* RNAi and assessed their numbers and distribution patterns. Immunostaining with an anti-tyrosine hydroxylase (TH) antibody revealed a significant reduction in the number of DA neurons in response to both pan-neuronal and type I NB-specific KD of *Baf* compared to that in the control group (Fig. 3e, f). On the other hand, type II NB-specific KD of *Baf* had no effect on either the number or distribution pattern of DA neurons (Fig. 3e, f). We also used the Q-system (*OK371*-QF2/+;QUAS-*mCD8GFP*/+) to label glutamatergic neurons in fly brains expressing *Baf* RNAi. Similar to DA neurons, the number of glutamatergic neurons in type I NBs was significantly decreased in fly brains pan-neuronally expressing *Baf* RNAi and those expressing *Baf* RNAi in type I NBs, but not in those expressing *Baf* RNAi in type II NBs (Fig. 3g, h). We then examined the number of other types of neurons, such as GABAergic, cholinergic, and serotonergic neurons, in adult brains pan-neuronally expressing *Baf* RNAi by immunostaining. Due to the high population density of GABAergic and cholinergic neurons in the central brain, we specifically counted the numbers of these neurons that were located near brain regions, such as the AMMC and SEZ, primarily associated with the subset of neurons comprising the neural circuit, which is responsible for grooming behavior^78,79^. Consequently, we assessed the numbers of these neurons located in the aforementioned regions of the *Baf* KD adult *Drosophila* brains and compared them to those in the control group. However, there were no significant changes in the numbers of GABAergic, cholinergic, or serotonergic neurons between the *Baf* KD and control brains (Supplementary Fig. 3f, g). Taken together, these data demonstrate that *Baf* KD impairs the formation of type I NB lineages and causes a substantial reduction in the number of excitatory neurons, such as DA and glutamatergic neurons, in adult brains.

### *Baf* KD in type I NBs induces distinct behavioral abnormalities in adults

**Fig. 4:**
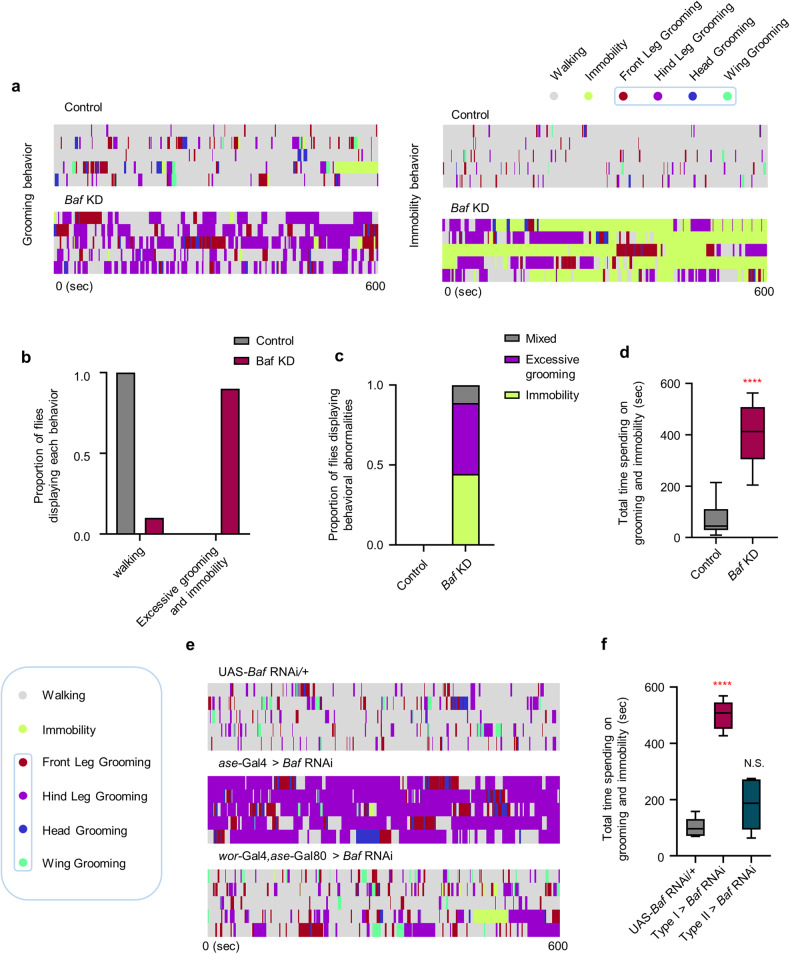
Behavioral abnormalities in adult flies lacking *Baf*. **a** Representative ethograms showing the distinct behaviors of control flies (*elav*-Gal4/+) and *Baf* KD flies (*elav*-Gal4/UAS-*Baf* RNAi). Different colors are used to distinguish different aspects of fly behavior. *n* = 5. **b** The proportion of control flies (*elav*-Gal4/+) or *Baf* KD flies (*elav*-Gal4/UAS-*Baf* RNAi) exhibiting aberrant behavior. The number of flies tested was as follows: Control = 5 flies and *Baf* KD = 5 flies. **c** Stacked bar graph showing the proportions of distinct behavioral abnormalities in control flies (*elav*-Gal4/+) or *Baf* KD flies (*elav*-Gal4/UAS-*Baf* RNAi). The number of flies tested was as follows: Control = 5 flies and *Baf* KD = 5 flies. **d** Box-and-whisker plots representing the 10th–90th percentiles with the mean value (horizontal line) of total time spent on grooming and immobility (*elav*-Gal4/+ or *elav*-Gal4/UAS-*Baf* RNAi) for 10 min. ****P < 1.0 × 10^−4^ by Student’s t-test; *n* = 5 flies. **e** Representative ethograms showing distinct behaviors of flies expressing the indicated genotypes (UAS-*Baf* RNAi/+, *ase*-Gal4/+;UAS-*Baf* RNAi/+, and *wor*-Gal4,*ase*-Gal80/+;UAS-*Baf* RNAi/+). Different colors are used to distinguish different aspects of fly behavior. *n* = 5. **f** Box-and-whisker plots representing the 10th–90th percentiles with the mean value (horizontal line) of total time spent on grooming and immobility in flies expressing the transgenes indicated in (**e**) for 10 min. N.S., not significant; ****P < 1.0 × 10^−4^ by one-way ANOVA with Tukey’s post hoc test; *n* = 5 flies.

Next, we explored the functional consequences of the aforementioned defects in the NB lineage formation caused by *Baf* KD. To this end, we characterized the behavioral phenotypes of adult flies expressing *Baf* RNAi driven by *elav*-Gal4. First, we observed that the majority (75%) of flies expressing *Baf* RNAi exhibited abnormal wing postures (droopy or held-up wing) (Supplementary Fig. 4a). The flies expressing *Baf* RNAi also displayed two additional behavioral abnormalities, characterized as excessive grooming and freezing-like behavior (termed “immobility behavior”) (Fig. 4a). To better characterize these unique behavioral abnormalities, we placed an individual male fly in a circular arena and monitored its movement for 10 min. The *Baf*-deficient flies exhibited a mixture of these two behavioral abnormalities. We considered a fly to be exhibiting a particular abnormal behavior when that behavior was observed for greater than one-third of the total recording time. In severe cases, flies exhibited both behavioral abnormalities, and these flies were labeled “mixed”. Notably, the majority of the flies lacking *Baf* exhibited aberrant behaviors (Fig. 4b, c). In line with these findings, adult flies pan-neuronally expressing *Baf* RNAi exhibited significantly increased total time spent on grooming and immobility compared to the control flies (Fig. 4d). Largely due to these unique behavioral abnormalities, flies expressing *Baf* RNAi barely walked and thus showed a reduced mean velocity (Supplementary Fig. 4b–d).

We next examined whether the observed behavioral abnormalities in *Baf*-deficient flies are primarily attributed to the neurodevelopmental defects that were characterized above prior to the maturation of the nervous system in the adult stage. To this end, we conditionally knocked down *Baf* before or after the eclosion of adult flies using Gal80^TS^, which is a temperature-sensitive negative regulator of Gal4 [temporal and regional gene expression targeting (TARGET) system]^80^, and assessed the aberrant behaviors of the adult flies. Conditional *Baf* KD before the eclosion of adult flies induced significant increases in the total time spent on grooming and immobility, whereas conditional *Baf* KD during the adult stage did not induce significant changes in grooming or immobility (Supplementary Fig. 4e). Consistently, we found that *Baf* KD in differentiated mature neurons or glial cells driven by *nSyb*- and *repo*-Gal4, respectively, did not induce behavioral abnormalities (Supplementary Fig. 4f, g). We further examined whether *Baf* KD in type I NBs recapitulates the unique behavioral abnormalities caused by the *elav*-Gal4-driven KD of *Baf*. We found that *Baf* KD in type I NBs, but not in type II NBs, markedly increased the behavioral abnormalities (Fig. 4e, f). Collectively, these data indicate that *Baf* deficiency in type I NBs during neurodevelopment prior to nervous system maturation leads to distinct behavioral abnormalities in the adult stage.

### Transcriptional downregulation of tsh, which interacts with beta-catenin, caused by *Baf* deficiency in type I NBs is associated with behavioral abnormalities in adult flies

**Fig. 5:**
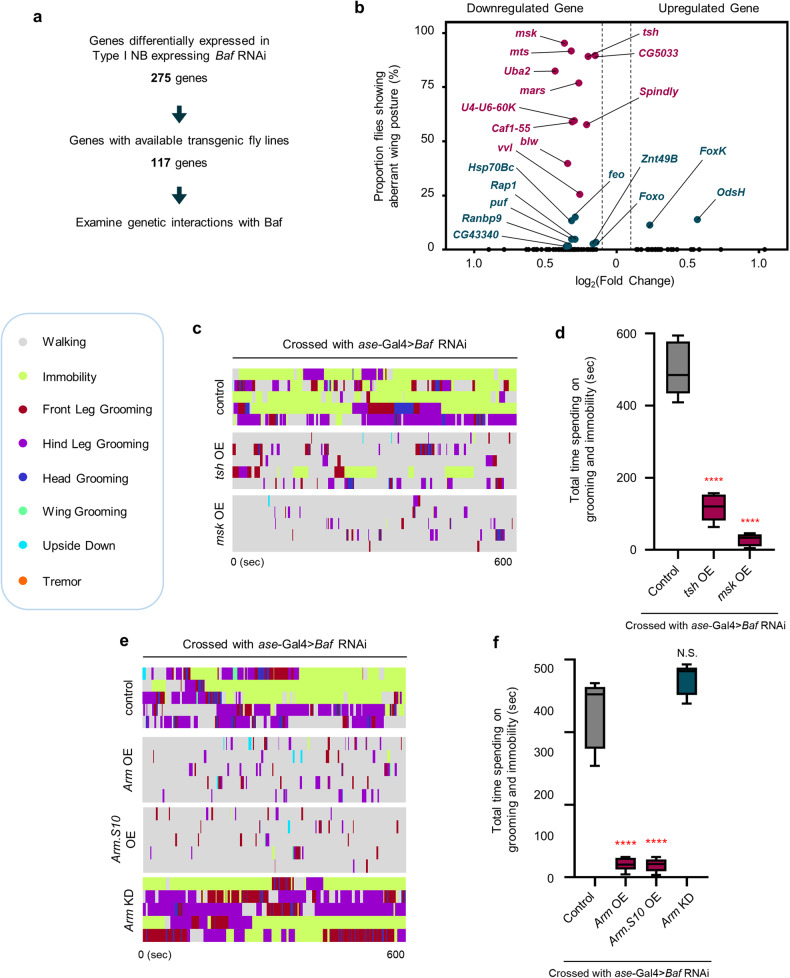
Identification of tsh, which interacts with beta-catenin, as a downstream mediator of *Baf* KD-induced behavioral abnormalities in adult flies. **a** The overall workflow for identifying downstream mediators of Baf-dependent regulation of type I NB-specific neurodevelopment. **b** Scatter dot plot showing the proportion of flies exhibiting aberrant wing postures induced by type I NB-specific expression of up- or downregulated DEGs that were identified in type I NBs. *n* ≥ 50 flies. Every scattered dot in the graph indicates KD of an indicated gene in type I NBs. Among these dots, red dots, but not turquoise dots, showed behavioral abnormalities in the adult stage (see Figure S5b for details). **c** Representative ethograms showing distinct behaviors of adult flies expressing transgenes as follows (*ase*-Gal4/+;UAS-*Baf* RNAi/+, *ase*-Gal4*/*UAS-*tsh*;UAS-*Baf* RNAi/+, and *ase*-Gal4/UAS-*msk*;UAS-*Baf* RNAi/+). Different colors are used to distinguish different aspects of fly behavior. *n* = 5. **d** Box-and-whisker plots representing the 10th–90th percentiles with the mean value (horizontal line) of total time spent on grooming and immobility in flies expressing the transgenes indicated in (**c**) for 10 min. N.S., not significant; ****P < 1.0 × 10^−4^ by one-way ANOVA with Tukey’s post hoc test; *n* = 5 flies. **e** Representative ethograms showing distinct behaviors of adult flies expressing transgenes as follows (*ase*-Gal4/+;UAS-*Baf* RNAi/+, *ase*-Gal4/UAS-*Arm*;UAS-*Baf* RNAi/+, UAS-*Arm*.*S10*/+;*ase*-Gal4/+;UAS-*Baf* RNAi/+, and *ase*-Gal4/+;UAS-*Baf* RNAi/UAS-*Arm* RNAi). Different colors are used to distinguish different aspects of fly behavior. *n* = 5. **f** Box-and-whisker plots representing the 10th–90th percentiles with the mean value (horizontal line) of total time spent on grooming and immobility in flies expressing the transgenes indicated in (**e**) for 10 min. N.S., not significant; ****P < 1.0 × 10^−4^ by one-way ANOVA with Tukey’s post hoc test; *n* = 5 flies.

We next investigated the molecular mechanism by which *Baf* KD-mediated transcriptional changes in type I NBs during neurodevelopment contribute to acquired behavioral abnormalities. To this end, we performed a series of genetic screenings that involved knocking down and overexpressing candidate genes that were identified as DEGs in type I NBs in our scRNA-seq data. Among the 275 DEGs (Supplementary Table 3), there were available transgenic fly lines that corresponded to 117 of the genes (Fig. 5a). As an initial screen, we individually overexpressed 30 upregulated genes and knocked down 87 downregulated genes in type I NBs and examined whether these flies exhibited aberrant wing postures similar to those of *Baf* KD flies (Supplementary Fig. 4a). Our initial screening revealed that aberrant wing postures were induced by knocking down 19 of the downregulated genes (*teashirt* [*tsh*], *Spindly*, *bellwether* [*blw*], *moleskin* [*msk*], *CG5033*, *Chromatin assembly factor 1 p55 subunit* [*Caf1*-*55*], *ventral veins lacking* [*vvl*], *microtubule star* [*mts*], *Ubiquitin*-*like activating enzyme 2* [*Uba2*], *U4-U6 small nuclear riboprotein factor 60K* [*U4-U6-60K*], *mars*, *fascetto* [*feo*], *Heat-shock-protein-70Bc* [*Hsp70Bc*], *Rap1 GTPase* [*Rap1*], *puffyeye* [*puf*], *Zinc transporter 49B* [*Znt49B*], *Foxo*, *Ranbp9* and *CG43340*) or by overexpressing 2 of the upregulated genes (*Forkhead box K* [*FoxK*] and *Ods-site homeobox* [*OdsH*]) in type I NBs (red and turquoise dots in Fig. 5b). We further evaluated whether downregulated 19 genes inducing wing posture problems in adult flies were correlated with heterochromatin remodeling/anchoring by conducting GO analysis of these genes compared to the other genes in the group of 117 genes. We found that among the GO terms specifically annotated to the 19 genes, those related to chromosome organization, such as chromosome segregation and nucleosome mobilization, were particularly enriched (Supplementary Fig. 5a). This enrichment suggested that the reduced gene expression may contribute to defective heterochromatin anchoring in type I NBs lacking *Baf* (Supplementary Fig. 5a).

We subsequently performed successive screening to examine whether genetic modulation of these 21 genes can induce behavioral abnormalities in the adult stage. Among the 21 genes, KD of 11 downregulated genes (*tsh*, *Spindly*, *blw*, *msk*, *CG5033*, *Caf1-55*, *vvl*, *mts*, *Uba2*, *U4-U6-60K*, and *mars*) in type I NBs induced aberrant behaviors in the adult flies (Fig. 5b (red dots) and Supplementary Fig. 5b) compared to the controls. Notably, increased behavioral abnormalities comparable to those induced by *Baf* KD were observed only in flies expressing *tsh* RNAi or *Spindly* RNAi (Supplementary Fig. 5c). We further examined whether genetic modulation of these 11 genes can suppress *Baf* KD-induced behavioral abnormalities. Among the 11 downregulated genes, only 4 of these genes had available overexpression transgenic fly lines. Overexpression of *tsh* or *msk* significantly suppressed *Baf* KD-induced behavioral abnormalities, whereas overexpression of *Caf1*-*55* or *mts* did not induce noticeable changes in the total time spent on grooming and immobility (Fig. 5c, d and Supplementary Fig. 5d, e), suggesting that Baf-dependent transcriptional control of *tsh* and *msk* is crucial for type I NB-specific neurodevelopment. We concluded that, between tsh and msk, tsh is a more potent factor in mediating Baf-dependent control of NB lineage development since KD of *tsh*, but not KD of *msk*, induced behavioral abnormalities comparable to those induced by KD of *Baf* (Supplementary Fig. 5b and see “Discussion” for details).

We next explored additional molecules/signaling pathways that could contribute to the Baf-dependent control of NB lineage development by interacting with tsh and/or msk. To this end, we examined the protein‒protein interaction (PPI) networks of tsh and msk using the Search Tool in the Retrieval of Interacting Genes/Proteins (STRING) web database and identified Armadillo [Arm], a *Drosophila* homolog of beta-catenin in the Wnt signaling pathway, as a factor that interacts with both tsh and msk. Previous studies reported that the zinc finger TF tsh binds to the C-terminus of Arm and is associated with trunk-specific modulation of the Wnt/beta-catenin pathway in *Drosophila* embryos^81,82^ and that msk, which is a *Drosophila* homolog of importin-7, induces the nuclear transport of Arm to regulate the Wnt signaling pathway^83^.

Given the well-characterized functions of the Wnt/beta-catenin pathway in the regulation of neural cell fate determination^84,85^, we speculated that Arm could contribute to Baf-dependent neurodevelopment through its interaction with tsh and/or msk. To experimentally validate this speculation, we first evaluated whether genetic disruption of *Arm* in type I NBs induces behavioral abnormalities in adult flies similar to those induced by *Baf* KD. Compared with *Baf* KD, *Arm* KD increased the total time spent on grooming and immobility (Fig. 4e, f and Supplementary Fig. 5f, g), which was consistent with our speculation. We then examined whether overexpression of *Arm* can ameliorate *Baf* KD-induced behavioral abnormalities. To this end, we individually overexpressed *Arm* or *Arm*.*S10*, a constitutively active form of Arm^86^, or knocked down *Arm* in type I NBs expressing *Baf* RNAi, and evaluated behavioral patterns in the adult stage. Notably, overexpressing *Arm* or *Arm*.*S10* significantly decreased the total time of *Baf* KD-induced behavioral abnormalities (Fig. 5e, f), which further validates our speculation. On the other hand, KD of *Arm* did not cause any noticeable changes in *Baf* KD-induced behavioral abnormalities (Fig. 5e, f), suggesting that *Baf* KD-induced behavioral abnormalities are already too severe to be further impaired.

### Transcriptional downregulation of tsh, which interacts with beta-catenin, caused by *Baf* deficiency in type I NBs is responsible for the type I NB lineage development

**Fig. 6:**
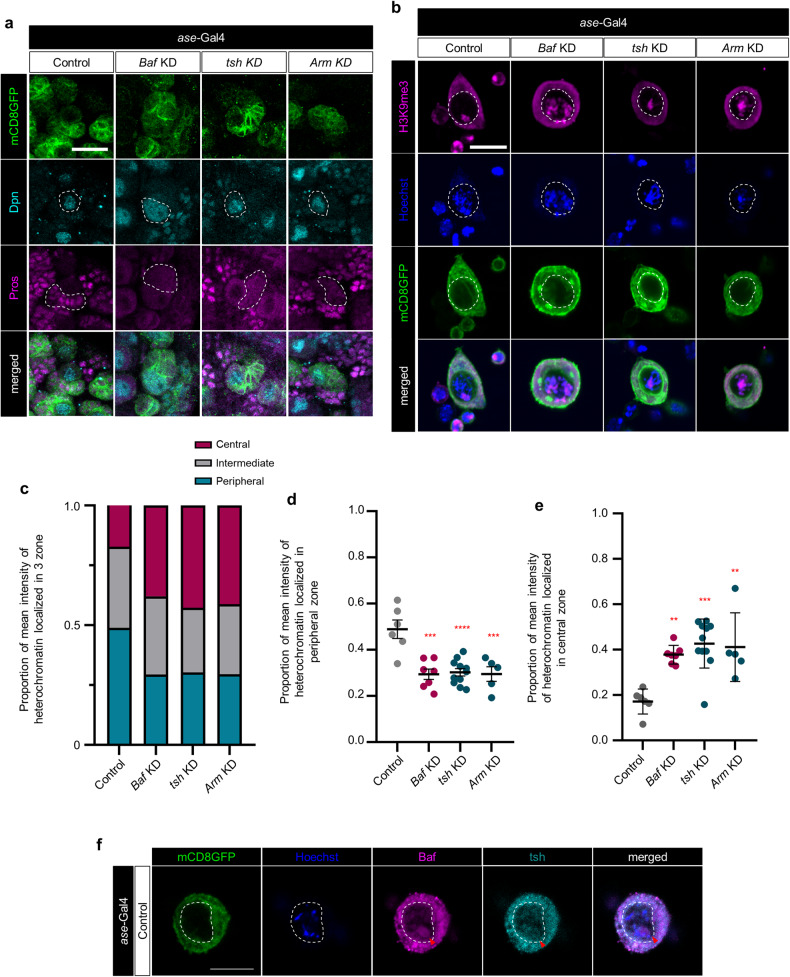
Characterization of the defects in heterochromatin anchoring and the formation of type I NB lineages induced by KD of *tsh* or *Arm* in type I NBs. **a** Representative images of immunostaining for Dpn (teal) and Pros (magenta) in control type I NBs and type I NBs expressing *Baf* RNAi (*Baf* KD), *tsh* RNAi (*tsh* KD), and *Arm* RNAi (*Arm* KD) in brains of third-instar larvae (*ase*-Gal4/+; UAS-*mCD8GFP*/+, *ase*-Gal4/+;UAS-*Baf* RNAi*/*UAS-*mCD8GFP*, *ase*-Gal4/+;UAS-*tsh RNAi/*UAS-*mCD8GFP*, and *ase*-Gal4/+;UAS-*Arm* RNAi/UAS-*mCD8GFP*). The white dashed lines indicate the outlines of NBs (second row) and neural progenies (third row) (Scale bar, 20 μm). **b** Representative images of immunostaining for H3K9me3 (magenta) with Hoechst staining in cultured primary control type I NBs and type I NBs expressing *Baf* RNAi (*Baf* KD), *tsh* RNAi (*tsh* KD), and *Arm* RNAi (*Arm* KD) that were isolated from the brains of third-instar larvae (*ase*-Gal4/+; UAS-*mCD8GFP*/+, *ase*-Gal4/+;UAS-*Baf* RNAi*/*UAS-*mCD8GFP*, *ase*-Gal4/+;UAS-*tsh* RNAi*/*UAS-*mCD8GFP*, and *ase*-Gal4/+;UAS-*Arm* RNAi/UAS-*mCD8GFP*). NBs were visualized by expression of the fluorescent plasma membrane marker protein mCD8GFP. The white dashed lines in the panels show the outlines of the inner nuclear membrane (Scale bar, 10 μm). **c** Stacked bar graph showing the proportion of mean intensity of heterochromatin localized in three zones of cultured primary type I NBs expressing the transgenes indicated in (**b**). The number of type I NBs tested was as follows: Control = 6, *Baf* KD = 7, *tsh* KD = 11, and *Arm* KD = 5. **d** Scatter dot plots displaying the proportion of mean intensity of heterochromatin localized in the peripheral zone of type I NBs expressing the transgenes indicated in (**b**). ***P < 1.0 × 10^−3^; ****P < 1.0 × 10^−4^ by one-way ANOVA with Tukey’s post hoc test; error bars, mean ± SEM. The number of type I NBs tested was as follows: Control = 6, *Baf* KD = 7, *tsh* KD = 11, and *Arm* KD = 5. **e** Scatter dot plots displaying the proportion of mean intensity of heterochromatin localized in the central zone of type I NBs expressing the transgenes indicated in (**b**). **P < 1.0 × 10^−2^; ***P < 1.0 × 10^−3^ by one-way ANOVA with Tukey’s post hoc test; error bars, mean ± SEM. The number of type I NBs tested was as follows: Control = 6, *Baf* KD = 7, *tsh* KD = 11, and *Arm* KD = 5. **f** Representative images of immunostaining for Baf (magenta) and tsh (teal) in cultured primary type I NBs that were isolated from the brains of third-instar larvae (*ase*-Gal4/+; UAS-*mCD8GFP*/+). NBs are visualized by expression of the fluorescent plasma membrane marker protein mCD8GFP. The white dashed lines in the panels show the outlines of the inner nuclear membrane (Scale bar, 10 μm).

We next examined whether KD of *tsh* or *Arm* recapitulates *Baf* KD-induced defects in the development of type I NB lineages. However, KD of *tsh* or *Arm* in type I NBs barely impaired the formation of type I NBs and their neural progenies (Supplementary Fig. 6a–c), indicating that KD of *tsh* or *Arm* alone is not sufficient to impair the symmetric/asymmetric division of type I NBs. Similarly, the expression of *msk* RNAi in type I NBs did not show any noticeable changes in the number of type I NBs and the formation of their neural progenies (Supplementary Fig. 6d, e). To further characterize the cellular defects caused by KD of *tsh* or *Arm* in type I NBs, we next examined whether KD of *tsh* or *Arm* in type I NBs leads to dysregulation of cell fate determinants in the neural progenies of type I NBs. Notably, the neural progenies derived from type I NBs lacking *tsh* or *Arm* showed reduced level of Pros, compared to those of control group (Fig. 6a), similar to what was observed in the case of *Baf* KD. These results collectively suggest that genetic disruption of *tsh* or *Arm* in type I NBs induces detrimental cellular changes in type I NBs and their neural progenies as well as dysregulation of cell fate determinants in the neural progenies, although these detrimental cellular changes are insufficient to disrupt the symmetric/asymmetric division of type I NBs.

We then wondered whether KD of *tsh* or *Arm*, which recapitulates *Baf* KD-induced defects in behavior and the formation of type I NB lineages also impairs the anchoring of heterochromatin to the INM. Quantification of the subnuclear positioning of heterochromatin (Supplementary Fig. 6f) was conducted as previously described^87,88^. Heterochromatin in type I NBs lacking *tsh* or *Arm* tended to localize to the center of the nucleus, comparable to that in type I NBs lacking *Baf* (Fig. 6b–e), suggesting that tsh and Arm are also needed to anchor heterochromatin to the INM, at least in type I NBs, rather than functioning downstream in the process of altered heterochromatin anchoring. To deepen our understanding of the molecular characteristics of key genes (Baf, tsh, and Arm) specifically in type I NBs, we examined the subcellular localization of these molecules in type I NBs by immunostaining. Notably, we detected Baf and tsh using their respective antibodies, revealing a pattern of colocalization in a region adjacent to DNA labeled by Hoechst staining within the nucleus of type I NBs (Fig. 6f). On the other hand, Arm was predominantly localized in the cytoplasmic region of type I NBs (Supplementary Fig. 6g), which is consistent with the previously described localization of Arm/beta-catenin in most cells in the absence of specific upstream cues^89,90^. We next examined whether there were any notable changes in the level or localization of key proteins between Arm, which is primarily localized in the cytoplasm, and the nuclear-localized Baf and tsh in type I NBs. However, the expression and localization of Arm in type I NBs did not noticeably change regardless of the presence of *Baf* or *tsh* (Supplementary Fig. 6h), and KD of *Arm* also did not induce alterations in the subcellular distribution of Baf and tsh in type I NBs compared to the control (Supplementary Fig. 6i). Overall, our immunohistochemistry data did not provide direct evidence of changes in the amount and/or localization of Arm and the nuclear-localized proteins Baf and tsh in type I NBs, despite the expected significant role of Arm in the formation of Type I NB lineages, as suggested by the genetic rescue data presented above (Fig. 5e, f).

To enhance our understanding of the mode-of-action of key genes engaged in anchoring heterochromatin to the INM in type I NBs, we examined whether KD of each key gene that colocalized to a region adjacent to heterochromatin in type I NBs affects the subcellular localization of the other key gene that colocalize to this region. In this regard, it should be noted that, in type I NBs lacking *Baf* showing altered subcellular localization of heterochromatin (Fig. 2a), immunostaining for tsh failed to detect signals for the endogenous tsh proteins (data not shown), consistent with our scRNA-seq data showing substantially decreased expression of *tsh*, but not *Arm*, in type I NBs (Supplementary Fig. 6j). Next, we investigated whether KD of *tsh* affects the subcellular localization of both Baf and heterochromatin in type I NBs. Notably, KD of *tsh* in type I NBs changed the subcellular localization of heterochromatin without affecting that of Baf (Supplementary Fig. 6k). Together with the pattern of tsh and Baf colocalization in the nucleus of type I NBs, these data collectively suggest that both Baf and tsh are required for anchoring heterochromatin to the INM in type I NBs, while the binding of heterochromatin to each of these proteins does not necessitate the presence of the other.

**Fig. 7:**
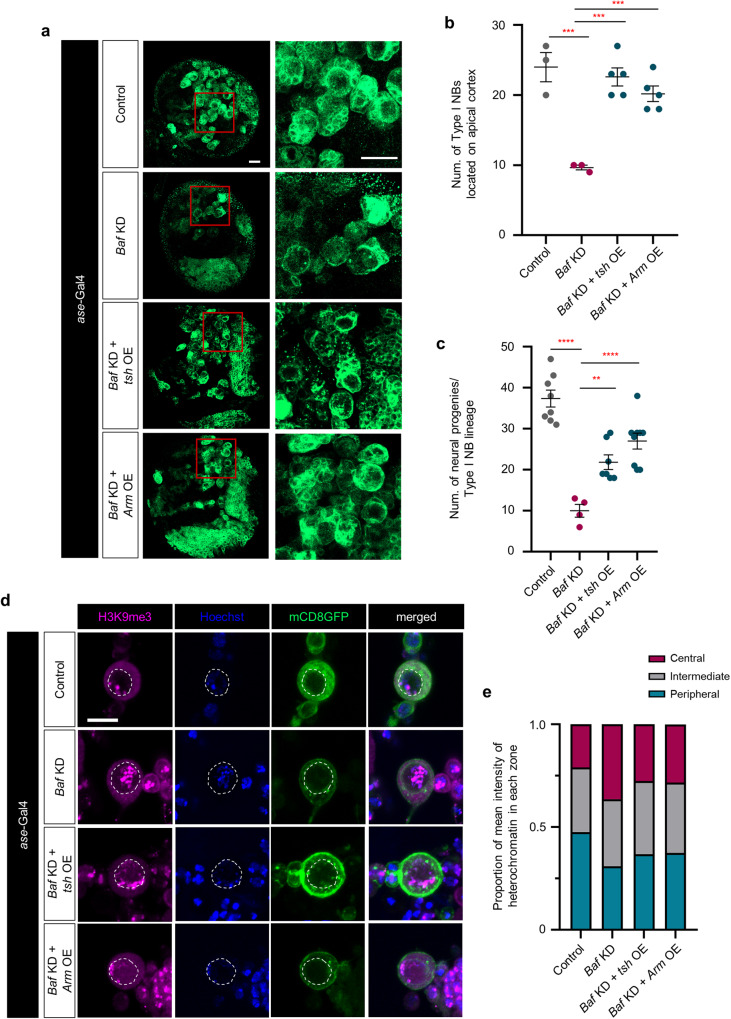
Characterization of the complementary roles of tsh and Arm in regulating the anchoring of heterochromatin and the formation of type I NB lineages. **a** Representative images of control type I NBs and type I NBs expressing *Baf* RNAi (*Baf* KD), *Baf* RNAi + *tsh* (*Baf* KD + *tsh* OE), and *Baf* RNAi + *Arm* (*Baf* KD + *Arm* OE) in the brains of third-instar larvae (*ase*-Gal4/+;UAS-*mCD8GFP*/+, *ase*-Gal4/+;UAS-*mCD8GFP*/UAS-*Baf* RNAi, *ase*-Gal4/UAS-*mCD4GFP*;UAS-*tsh*/UAS-*Baf* RNAi, and *ase*-Gal4/UAS-*Arm*;UAS-*mCD8GFP*/UAS-*Baf* RNAi). Magnified images of the red squares in the *upper* panels are presented in the *lower* panels (Scale bars, 20 μm). **b** Quantification of the number of type I NBs located in the apical cortex of third-instar larval brain lobes expressing the transgenes indicated in (**a**). ***P < 1.0 × 10^−3^ according to one-way ANOVA with Tukey’s post hoc test; error bars, mean ± SEM; the number of brain lobes tested was as follows: Control = 3 brain lobes, *Baf* KD = 3 brain lobes, *Baf* KD + *tsh* OE = 5 brain lobes, and *Baf* KD + *Arm* OE = 5 brain lobes. **c** Quantification of the number of neural progenies derived from type I NBs located in the apical cortex of third-instar larval brain lobes expressing the transgenes indicated in (**a**). **P < 1.0 × 10^−2^, ****P < 1.0 × 10^−4^ by one-way ANOVA with Tukey’s post hoc test; error bars, mean ± SEM; the number of NBs tested was as follows: Control = 8 NBs, *Baf* KD = 4 NBs, *Baf* KD + *tsh* OE = 7 NBs, and *Baf* KD + *Arm* OE = 9 brain lobes. **d** Representative images of cultured primary type I NBs isolated from the brains of third-instar larvae expressing the transgenes indicated in (**a**). The white dashed lines in the panels show the outline of the inner nuclear membrane (Scale bar, 10 μm). **e** Stacked bar graph showing the proportion of mean intensity of heterochromatin localized in three zones of cultured primary type I NBs expressing the genes indicated in (**a**). The number of type I NBs tested was as follows: Control = 12, *Baf* KD = 10, *Baf* KD + *tsh* OE = 14, and *Baf* KD + *Arm* OE = 9.

Finally, we wondered whether overexpression of *tsh* or *Arm* in type I NBs lacking *Baf* is capable of suppressing *Baf* KD-induced defects in the formation of type I NB lineages during neurodevelopment. Of note, overexpression of *tsh* or *Arm* markedly restored these numbers of type I NBs and their neural progenies impaired by *Baf* deficiency (Fig. 7a–c). Moreover, *Baf* deficiency altered the localization of heterochromatin in type I NBs, but overexpression of *tsh* or *Arm* restored the subnuclear position of heterochromatin to the peripheral region of the nucleus (Fig. 7d, e and Supplementary Fig. 7a, b). Furthermore, overexpression of *tsh* or *Arm* in type I NBs lacking *Baf* partially restored the expression level of Pros in the neural progenies derived from type I NBs (Supplementary Fig. 7c). Consistent with the finding that overexpression of *tsh* or *Arm* suppressed *Baf* KD-induced defects in the type I NB lineage during larval development, overexpression of *tsh* or *Arm* restored the number of DA neurons, which had been impaired by *Baf* deficiency (Supplementary Fig. 7d, e). Collectively, our genetic analyses identified tsh, which interacts with Arm, as a key downstream mediator of Baf in the development of type I NB cell lineages and as a crucial factor that complements the Baf-dependent control of heterochromatin anchoring.

## Discussion

In this study, we demonstrated that *Baf* deficiency in type I NBs of *Drosophila* causing dysregulation of heterochromatin anchoring leads to impairment of type I NB-specific neurodevelopment and subsequent behavioral abnormalities. Notably, among the DEGs in type I NBs that were identified in our scRNA-seq data, our genetic analyses revealed that tsh, a zinc-finger TF that interacts with beta-catenin in the Wnt signaling pathway, is a key downstream mediator of Baf in the regulation of NB lineages and a complementing factor of Baf-dependent control of heterochromatin anchoring. We also found that tsh colocalizes with Baf in a region adjacent to heterochromatin in type I NBs, which may provide valuable clues for understanding the mode-of-action underlying the cooperative regulation of heterochromatin anchoring to the INM by Baf and tsh in the development of type I NB lineages.

Notably, *Baf* KD caused prominent changes in transcriptional profiles, particularly in type I NBs, although *elav*-Gal4 drives the expression of *Baf* RNAi globally throughout the central nervous system. This cell type-specific effect of *Baf* KD can be explained by the low Baf protein expression specifically in type I NBs. Although we think that the level of Baf expression in the central nervous system of *Drosophila* larvae may not be high, judging from the relatively small detection of *Baf* mRNA levels in our scRNA-seq data and the generally weak intensity of immunostaining with an anti-Baf antibody (data not shown), we excluded the possibility of low Baf expression specifically in type I NBs based on a previous study that used an anti-Baf antibody to show ubiquitous expression of endogenous Baf throughout entire brains of larvae^32^. Instead, it seems more likely that functionally redundant molecules of Baf (e.g., other chromatin-binding proteins), downstream mediators of Baf (e.g., tsh and Arm), and/or interactors of Baf (e.g., LEM domain-containing proteins) may be more abundant/active in other cell types, increasing the sensitivity of type I NBs to *Baf* KD. In this regard, considering our findings (Fig. 2a) and the findings of previous studies showing that *Baf* deficiency fails to generate intact progenies through mitotic cell division, we carefully speculate that cell type-specific effects may be critically regulated by the presence and/or amounts of tsh to the level comparable to or even more important than those of Baf, which is thought to be ubiquitously expressed and has essential mitotic functions.

Interestingly, certain processes involved in mammalian neurogenesis share similarities with those involved in *Drosophila* neurogenesis. In particular, the key principle of neurogenesis (e.g., asymmetric division accompanied by a polarized distribution of cell fate determinants) appears to be evolutionarily conserved. In the mammalian neocortex, various types of neural progenitors, such as radial glial cells, short neural precursors, and outer radial glial cells, can be found. These progenitors undergo asymmetric division, allowing them to both self-renew and produce neurons/intermediate neural progenitors^91,92^. Among them, radial glial cells in mammals largely undergo asymmetric cell division, similar to type I NBs in *Drosophila*^93,94^. As in *Drosophila*, the fate of neural progenitor cells in mammals is governed by the spatial and temporal TFs, enabling these cells to give rise to diverse types of neural progeny^95,96^. However, our understanding of the upstream regulators or regulatory processes governing the sequential expression of TFs during mammalian development remains relatively less understood. To bridge this knowledge gap, the mechanistic insights from our study may be broadly applicable beyond *Drosophila* and could be extended to the mammalian system. Notably, Banf1, a mammalian homolog of *Baf*, functions as a chromatin-anchoring protein located within the inner nuclear membrane^26^. In addition, a previous study demonstrated that deficiencies in *Banf1* result in reduced expression of TFs that are associated with pluripotency in mouse embryonic stem cells and lead to defects in survival of both mouse and human embryonic stem cells^97^. Based on these findings, we speculated that Banf1 may also play an essential role in the development of neural progenitor cell lineages by mediating the subnuclear positioning of chromatin and regulating the transcription of genes that determine cell lineages. To experimentally validate this, scRNA-seq experiments and *Banf1*-deficient mouse studies should be conducted in a manner similar to our study. In addition to *Banf1*, a more focused study on *tshz1*, the mammalian counterpart of *tsh*, is needed to validate our findings in the mammalian context. Notably, previous research revealed that *tshz1* functions as a transcriptional regulator of genes that are involved in neurodevelopment^98,99^. According to a previous study, tshz1 is involved in the differentiation and radial migration of neural progenitor cells within the olfactory bulb^99^. It will be interesting to see whether our findings are applicable to this process. We hope that the application of our findings to mammalian systems in this respect will provide a new direction for understanding the cell type-specific regulation of neurogenesis via the control of heterochromatin anchoring by specific molecular machineries, which primarily consist of Banf1 and tshz1.

Among the DEGs that were identified in type I NBs, our genetic analyses revealed that the downregulation of *tsh* or *msk* may be related to the *Baf* KD-induced phenotypes. Type I NB-specific overexpression of these genes significantly suppressed *Baf* KD-induced behavioral abnormalities (Fig. 5c, d). However, we focused on *tsh* in our study because KD of *msk* induced a distinct behavioral phenotype, tremor, that was not observed in adult flies lacking *Baf* or *tsh* (Supplementary Fig. 5b). The occurrence of distinct behavioral defects induced by *msk* KD suggested that *msk* KD involves additional molecular mechanisms other than those involving Arm. We suspect that the mitogen-activated protein kinase (MAPK) signaling pathway is a potential target based on previous reports indicating that msk, a *Drosophila* homolog of importin-7, is involved in the translocation of activated MAPK into the nucleus^100,101^. Given the well-characterized roles of MAPK signaling in the determination of neural cell fate^102,103^, dysregulation of MAPK signaling by *msk* KD in type I NBs may lead to abnormal neurodevelopment and distinct behavioral abnormalities. Since overexpression of *msk* markedly suppressed the *Baf* KD-induced phenotypes, it will be interesting to determine whether MAPK signaling also participates in the Baf-mediated regulation of the type I NB lineage development in addition to beta-catenin.

In summary, our study provides evidence that the heterochromatin anchoring-dependent transcriptional regulation of specific gene(s) may be essential for the formation of neural progenitor cell lineages and subsequent neurodevelopment. Moreover, our data provide new mechanistic insight that tsh interacting with Arm, of which expression is regulated by Baf in a cell type-specific manner, colocalizes with Baf in a region adjacent to heterochromatin in type I NBs and can complement the role of Baf in the formation of NB lineages through its overexpression, under conditions of *Baf* deficiency. Our unique approach of combining scRNA-seq and a series of genetic analyses will inspire other studies in exploring the regulatory mechanisms involved in the development of neural progenitor cell lineages.

### Supplementary information

Supplementary Information
