# Supplementary material for: Baf-mediated transcriptional regulation of teashirt is essential for the development of neural progenitor cell lineages
Source: Exp Mol Med. 2024 Feb 19;56(2):422–40. doi: 10.1038/s12276-024-01169-3 (PMC10907700; doi:10.1038/s12276-024-01169-3)
Supplement: Supplementary file 1 — Supplementary Information [file 12276_2024_1169_MOESM1_ESM.pdf]

Supplementary Information

Supplementary Fig. 1 (continued)

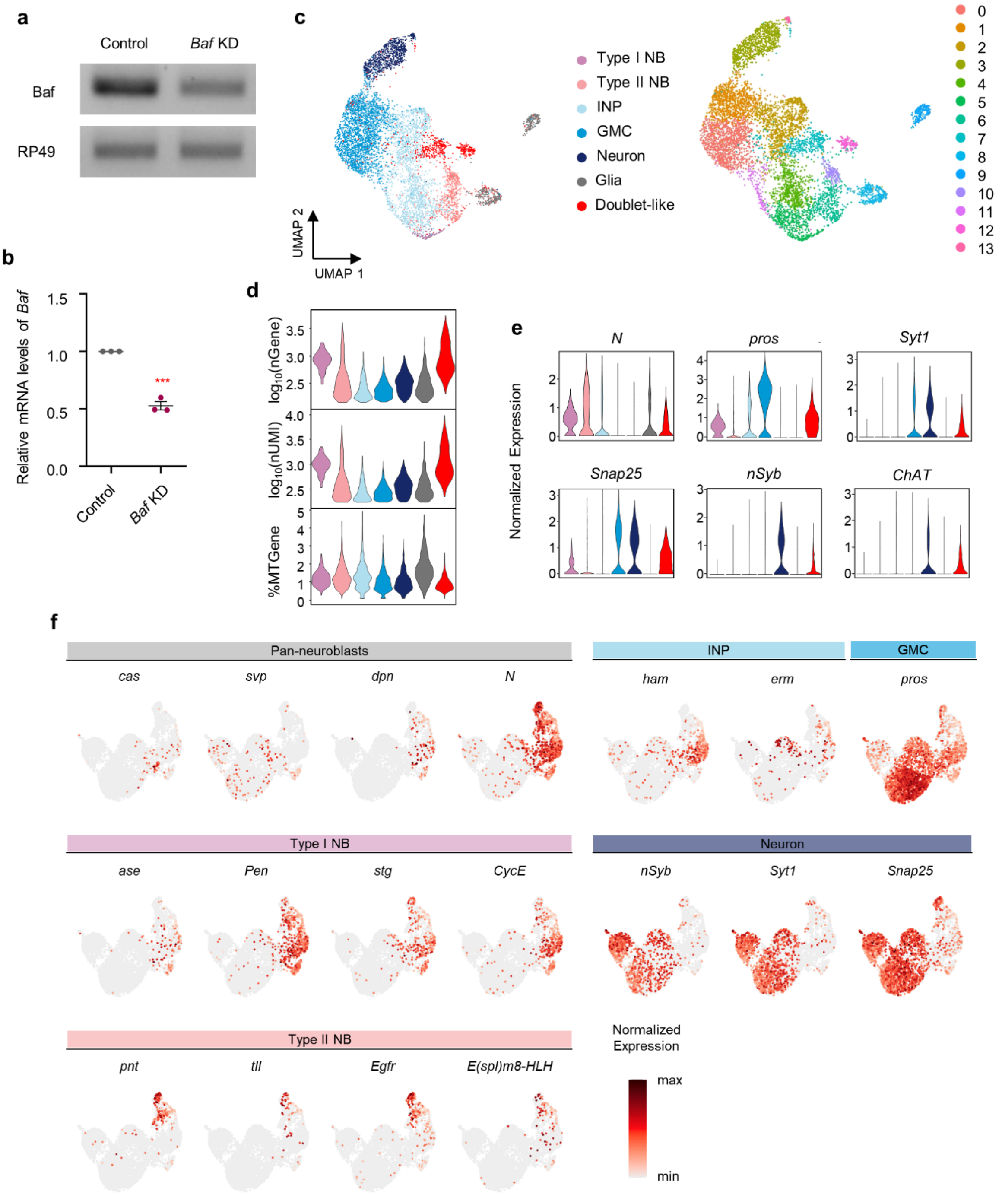

Supplementary Fig. 1

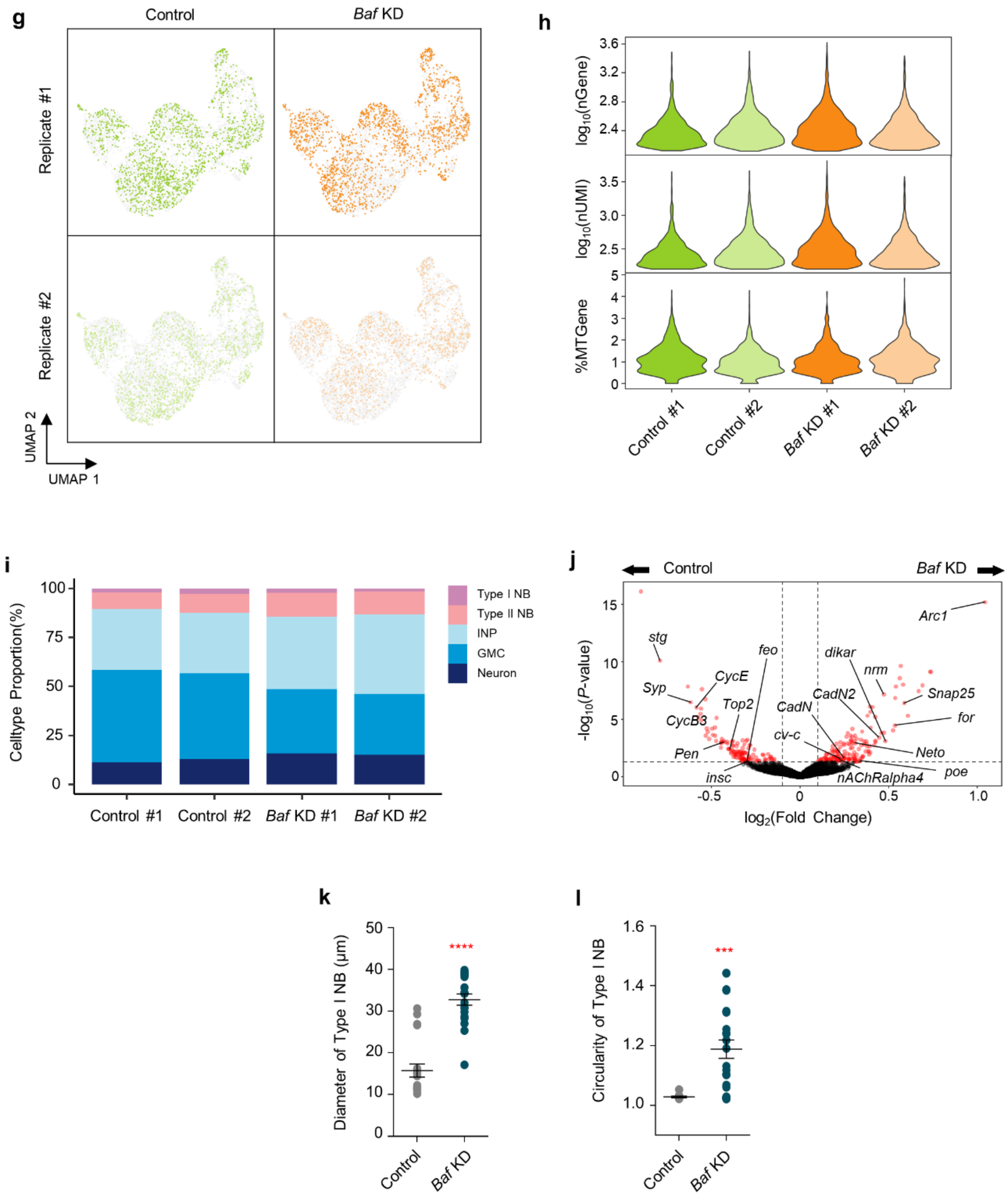

Supplementary Fig. 2

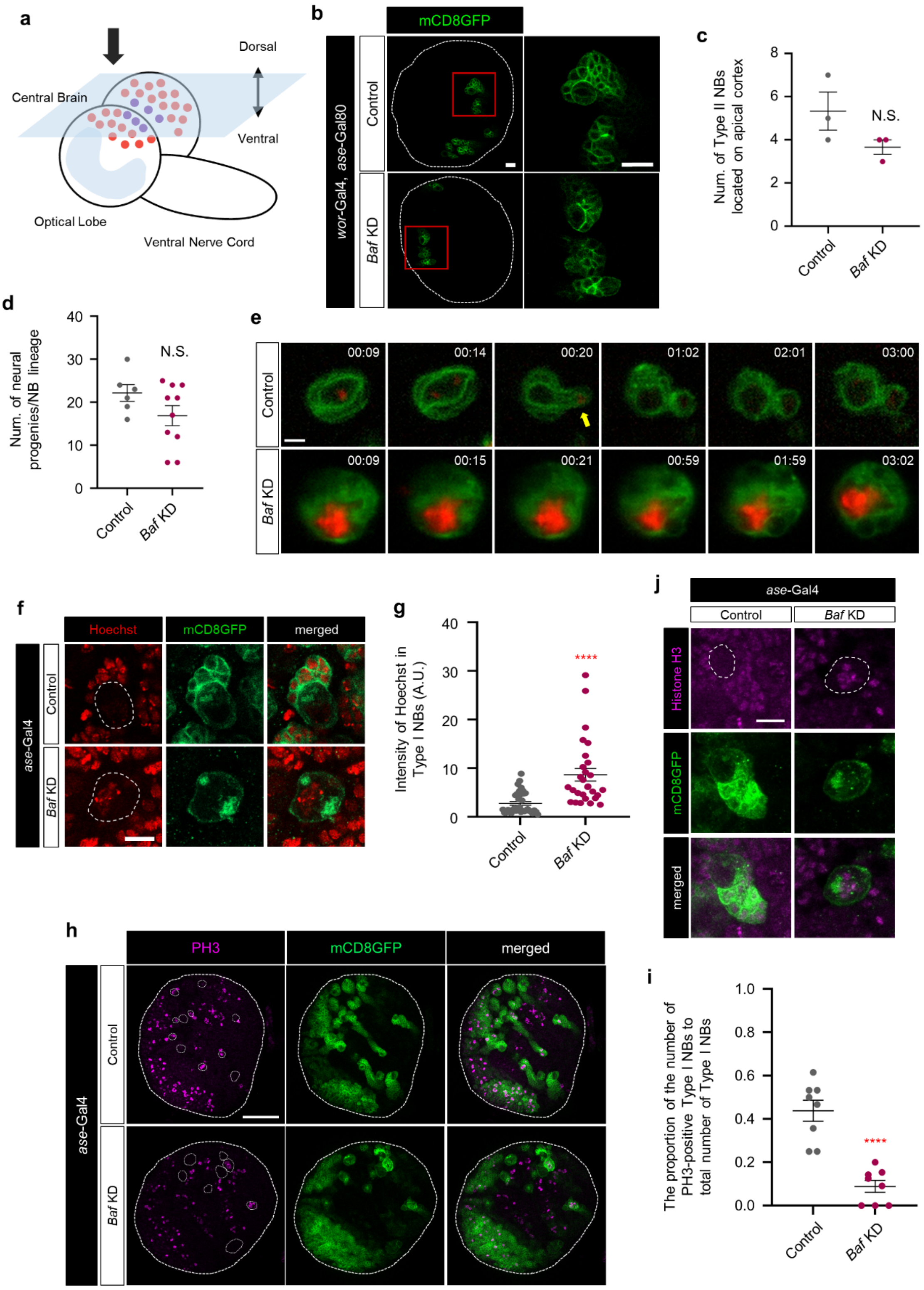

Supplementary Fig. 3 (continued)

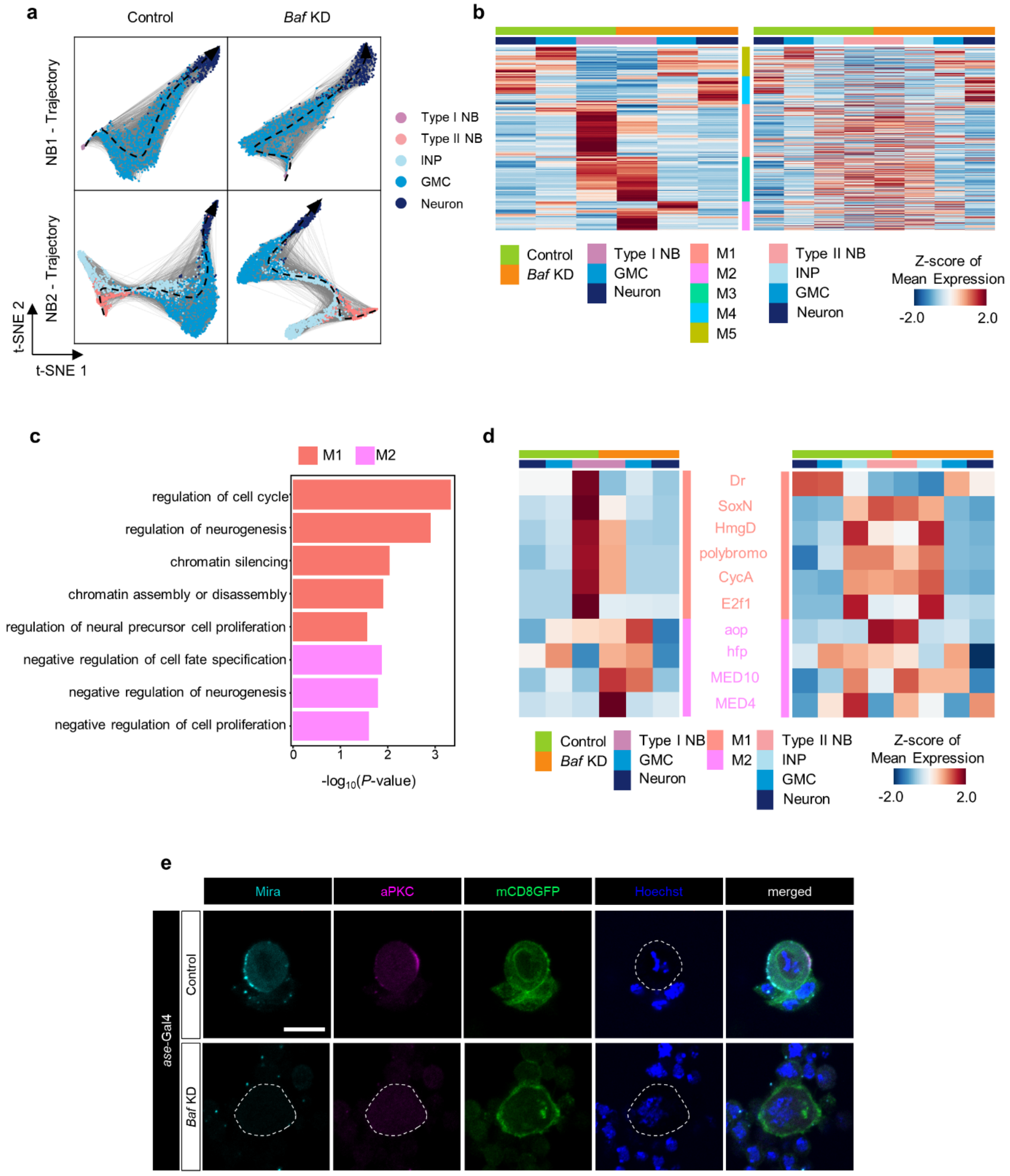

Supplementary Fig. 3

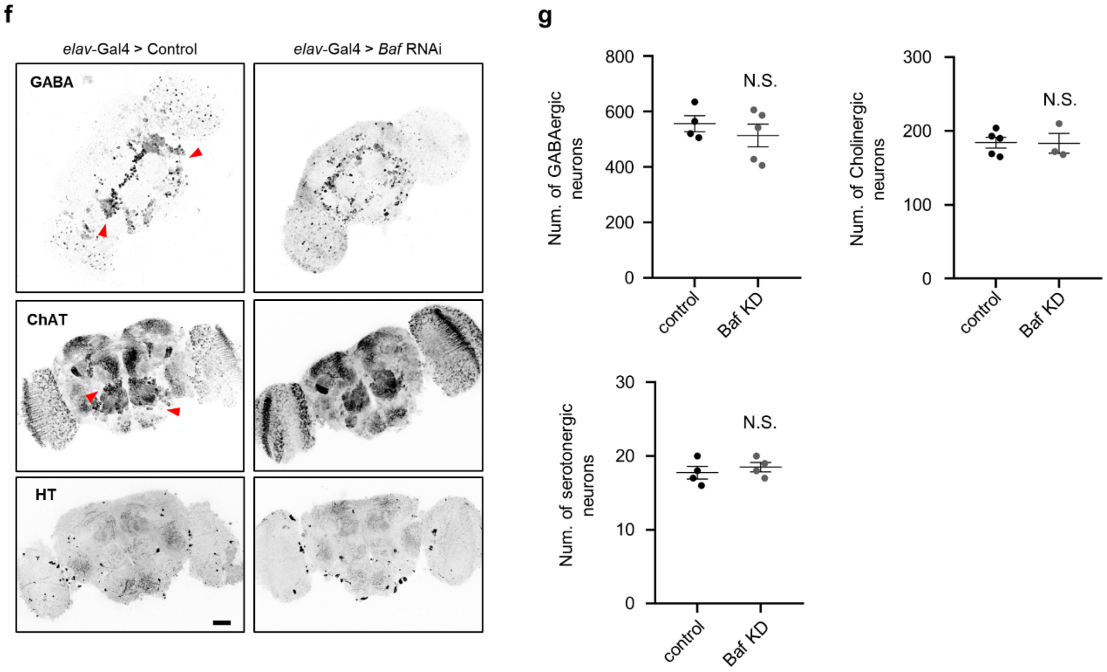

Supplementary Fig. 4

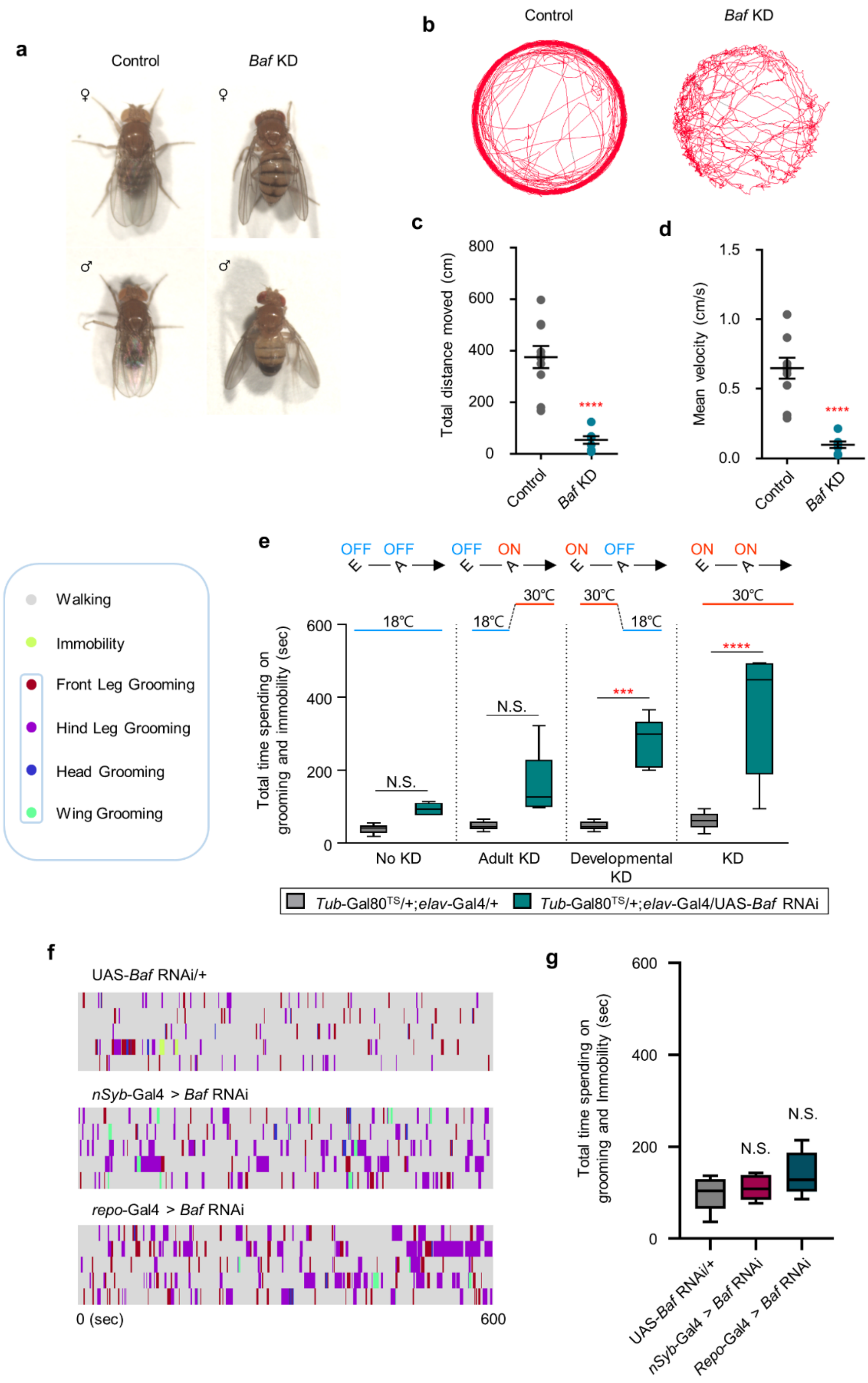

Supplementary Fig. 5

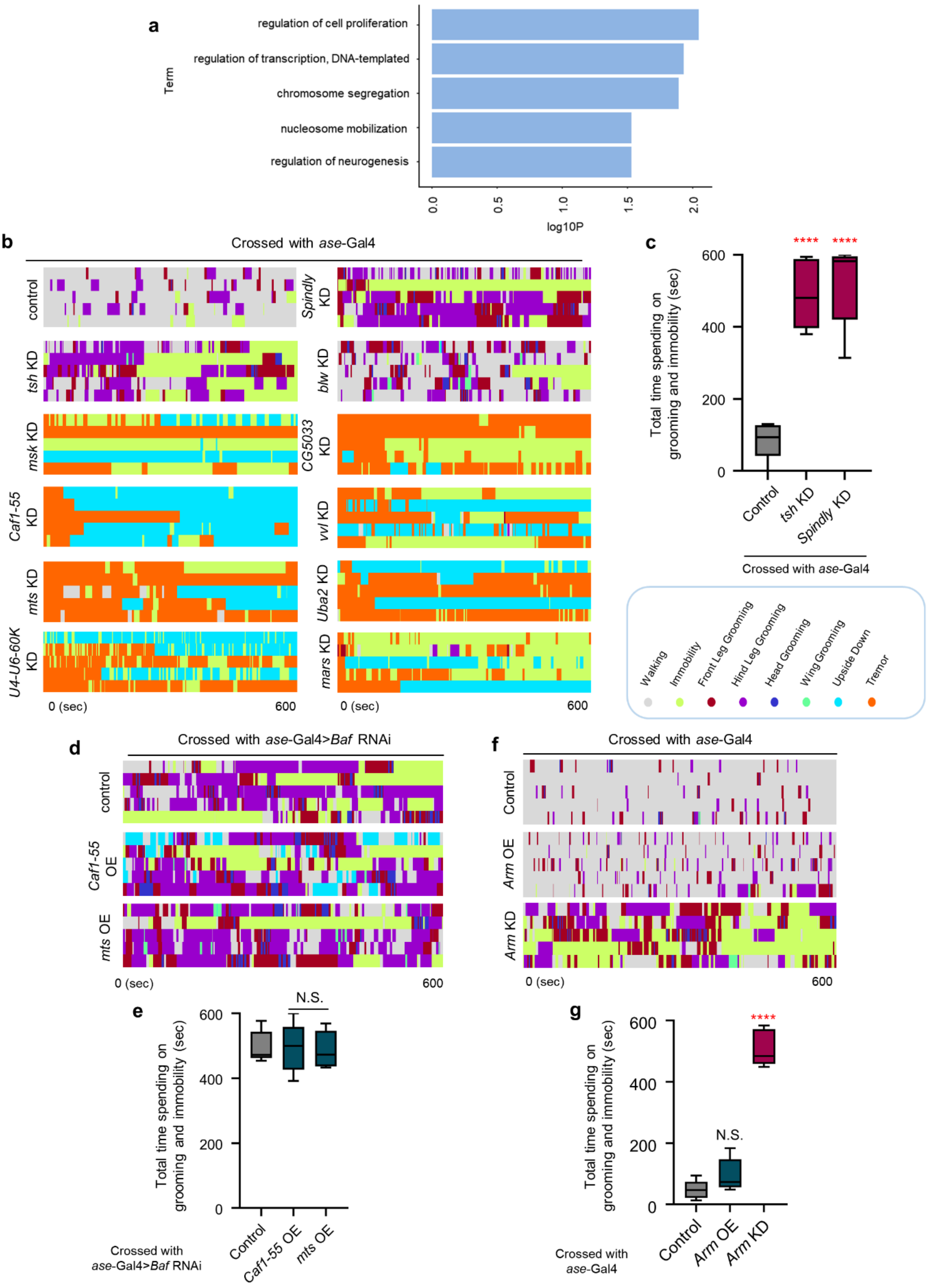

Supplementary Fig. 6 (continued)

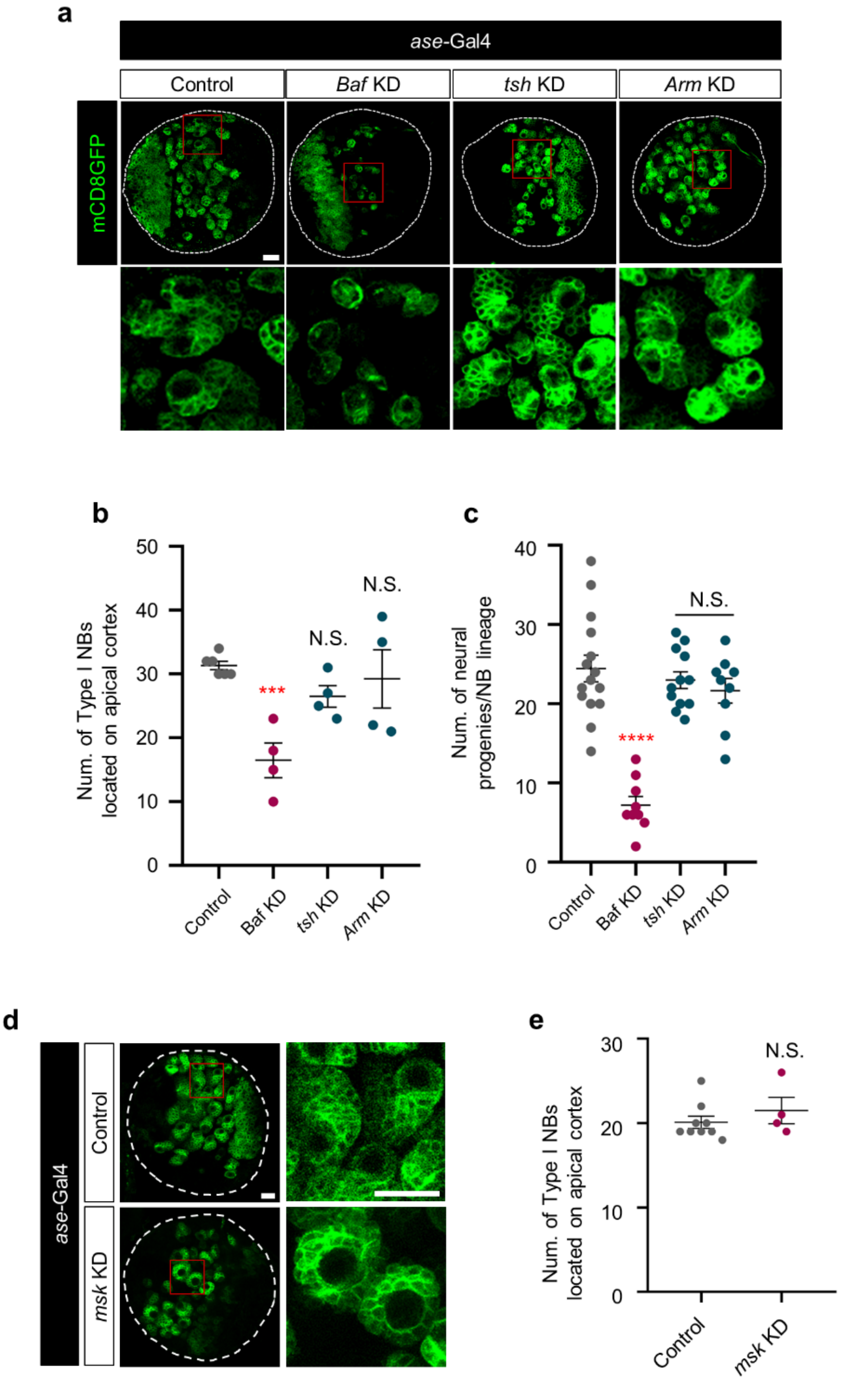

Supplementary Fig. 6

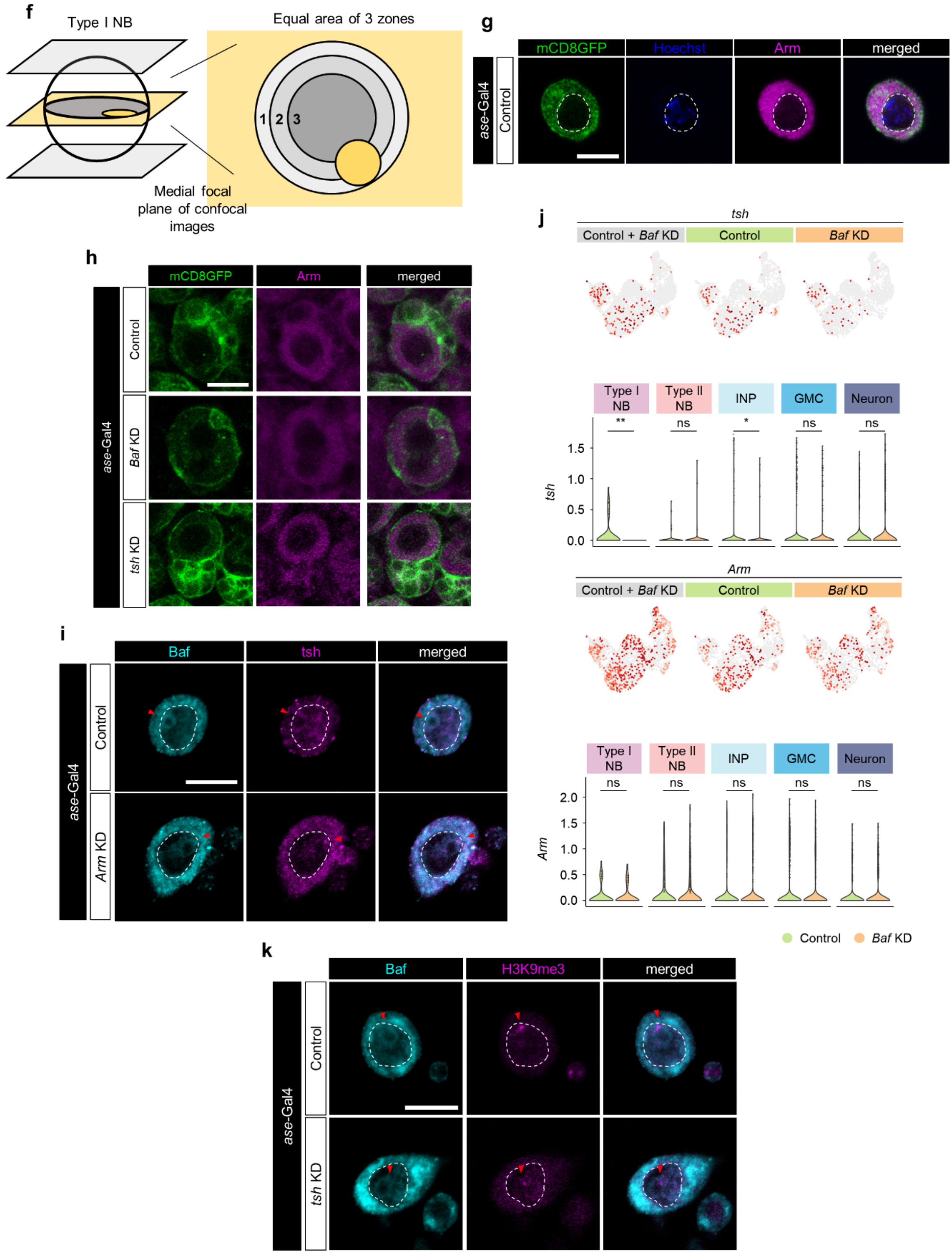

Supplementary Fig. 7

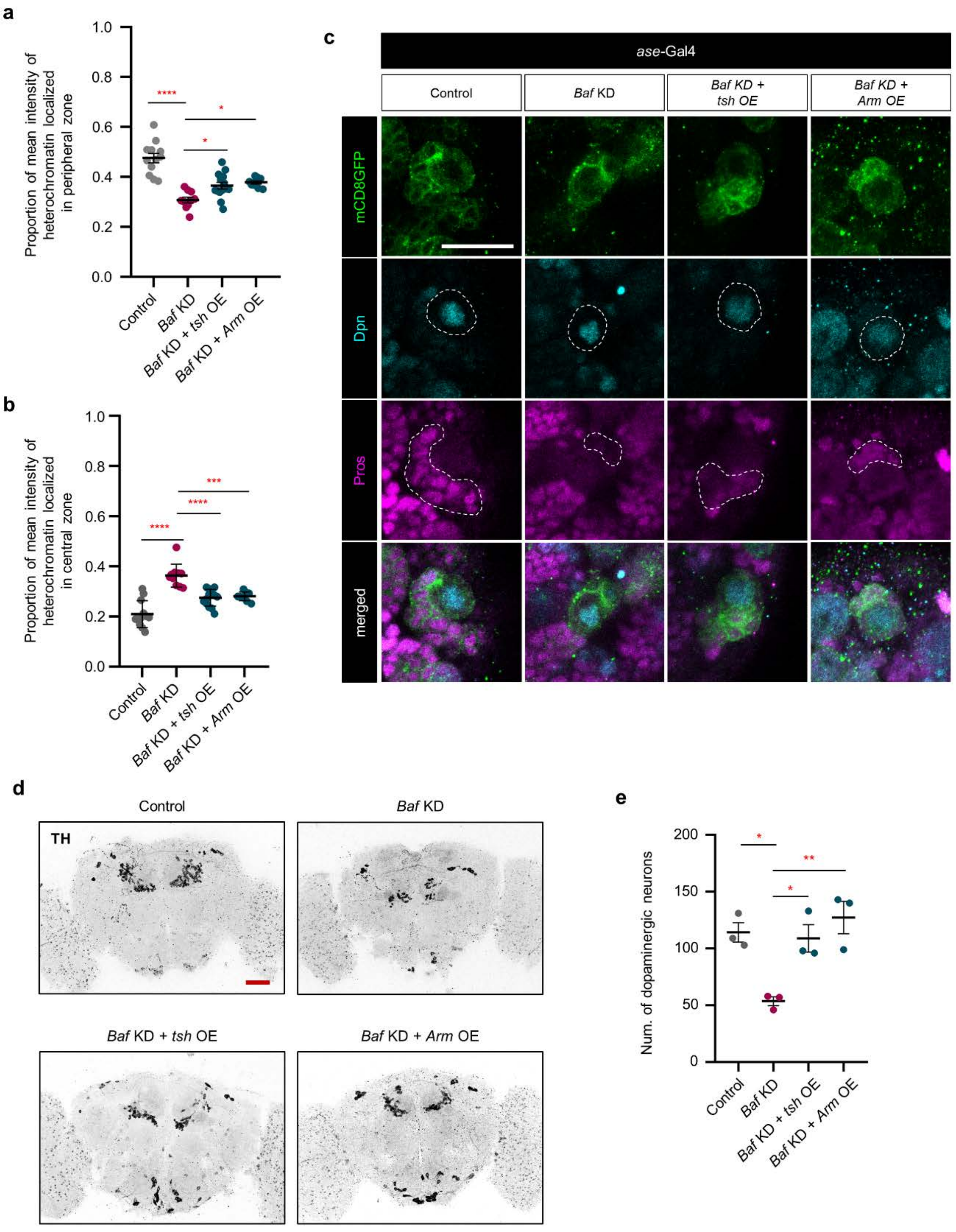

Supplementary figure legends

Supplementary Fig. 1. Single-cell transcriptional profiles of control and type I NBs lacking *Baf*

(a) Representative images of RT-PCR products showing mRNA level of *Baf* in adult fly heads expressing following genotypes: *elav*-Gal4/+, *elav*-Gal4/UAS-*Baf* RNAi.

(b) Quantification of band intensities of RT-PCR products showing mRNA level of *Baf* in adult fly heads expressing the transgenes indicated in (*a*). *** $P < 1.0 \times 10^{-3}$ according to Student's t-test; error bars, mean ± SEM; n = 3 independent experiments.

(c) UMAP plots showing all cells before removing doublet-like cells, which are individually colored according to annotated cell types (*left*) or unbiasedly generated clusters (*right*).

(d) Violin plots displaying the log-scaled number of detected genes (*top*), UMIs (*middle*), and the percentage of mitochondrial gene expressions (*bottom*). Colors of each diagram indicates annotated cell types in (*c*).

(e) Violin plots showing the expressions of major markers of each cell type, which are co-detected from doublet-like cell cluster. Colors of each diagram indicate annotated cell types indicates annotated cell types in (*c*).

(f) UMAP plots showing the normalized expressions of cell type-specific markers in individual neuronal cells excluding glial cells.

(g) UMAP plots showing information of all cells after removing the double-like cluster and glial cells in each sample replicates.

(h) Violin plots showing the log-scaled number of detected genes (*top*), UMIs (*middle*), and

the percentage of mitochondrial gene expressions (*bottom*) for each sample replicates.

(i) Stacked bar graph showing the fraction of cells following to annotated cell types in each sample replicates (*left*) and the fraction of cells in individual sample replicates classified into each cell types (*right*).

(j) Volcano plot illustrating the differentially expressed genes between control and *Baf* KD cells in type I NBs (red dots).

(k) Aligned dot plots showing the diameter of cultured primary control type I NBs (*ase*-Gal4/+;UAS-*mCD8GFP*,UAS-*H2A.mRFP*/+) and type I NBs lacking *Baf* (*ase*-Gal4/+;UAS-*mCD8GFP*,UAS-*H2A.mRFP*/UAS-*Baf* RNAi) isolated from the brains of third-instar larvae. **** $P < 1.0 \times 10^{-4}$ according to Student's t-test; error bars, mean ± SEM; the number of NBs was as follows: Control = 19 NBs, *Baf* KD = 19 NBs.

(l) Aligned dot plots showing the circularity of type I NBs expressing the transgenes indicated in (*k*). *** $P < 1.0 \times 10^{-3}$ according to Student's t-test; error bars, mean ± SEM; the number of NBs was as follows: Control = 11 NBs, *Baf* KD = 22 NBs.

Supplementary Fig. 2. Characterization of cellular defects in NBs lacking *Baf*

(a) A schematic diagram of the imaging NBs in third-instar larvae brain using confocal microscope. Blue plane indicates a focal plane of confocal image. Each dot indicates NBs (*red* – type I NBs, *purple* – type II NBs) located in the apical cortex of third-instar larval central brain.

(b) Representative images of control type II NBs (*top*, *wor*-Gal4,*ase*-Gal80/+;UAS-*mCD8GFP*,UAS-*H2A.mRFP*/+) and type II NBs expressing *Baf* RNAi (*bottom, wor*-Gal4,*ase*-Gal80/+;UAS-*mCD8GFP*,UAS-*H2A.mRFP*/UAS-*Baf* RNAi) in the brains of third-instar larvae. The white dashed lines indicate the outlines of the brain lobes. Magnified images of red squares in the *left* panels are presented in the *right* panels. (Scale bars, 20 μm.)

(c) Quantification of the number of type II NBs located in the apical cortex of third-instar larval brain lobes expressing the transgenes indicated in (*b*). N.S., not significant according to Student's t-test; error bars, mean ± SEM; the number of brain lobes tested was as follows: Control = 3 brain lobes, *Baf* KD = 3 brain lobes.

(d) Quantification of the number of the neural progenies derived from type II NBs located in the apical cortex of third-instar larval brain lobes expressing the transgene indicated in (*b*). N.S., not significant according to Student's t-test; error bars, mean ± SEM; the number of NBs tested was as follows: Control = 6 NBs, *Baf* KD = 10 NBs.

(e) Live imaging of cultured primary type I NBs (*top*, *ase*-Gal4/+;UAS-*mCD8GFP*,UAS-*H2A.mRFP*/+) and type I NBs expressing *Baf* RNAi (*bottom*, *ase*-Gal4/+;UAS-*mCD8GFP*,UAS-*H2A.mRFP*/UAS-*Baf* RNAi) isolated from the brains of third-instar larvae with time course. Imaged time is presented as hour:minute. The yellow arrow indicates the

budding of daughter cell. (Scale bar, 5 μm.)

(f) Representative Hoechst-stained images of control type I NBs (*top, ase*-Gal4/+;UAS-*mCD8GFP*/+) and type I NBs expressing *Baf* RNAi (*bottom, ase*-Gal4/+;UAS-*mCD8GFP*/UAS-*Baf* RNAi) in the brains of third-instar larvae. The white dashed lines indicate the outlines of NBs. Merged images for Hoechst and mCD8GFP are also presented. (Scale bar, 10 μm.)

(g) Quantification of Hoechst intensity (A.U., arbitrary unit) within nucleus of type I NBs expressing the transgenes indicated in (*f*). **** $P < 1.0 \times 10^{-4}$ according to Student's t-test; error bars, mean ± SEM; the number of NBs tested was as follows: Control = 35 NBs, *Baf* KD = 28 NBs.

(h) Representative images of immunostaining for PH3 (magenta) in control type I NBs and type I NBs expressing *Baf* RNAi in the brains of third-instar larvae. (*ase*-Gal4/+;UAS-*mCD8GFP*/+ and *ase*-Gal4/+;UAS-*mCD8GFP*/UAS-*Baf* RNAi). The white dashed lines indicate the outlines of brain lobes. Merged images for PH3 and mCD8GFP are also presented. (Scale bar, 50 μm.)

(i) Quantification of the proportion of the number of PH3-positive type I NBs to total number of type I NBs located in the apical cortex of third-instar larval brain lobes expressing the transgene indicated in (h). **** $P < 1.0 \times 10^{-4}$ according to Student's t-test; error bars, mean ± SEM; the number of brain lobes tested was as follows: Control = 8 brain lobes, *Baf* KD = 8 brain lobes.

(j) Representative images of immunostaining for histone H3 (magenta) in control type I NBs and type I NBs expressing *Baf* RNAi in the brains of third-instar larvae. (*ase*-Gal4/+;UAS-

mCD8GFP/+ and *ase*-Gal4/+;UAS-*mCD8GFP*/UAS-*Baf* RNAi). The white dashed lines indicate the outlines of brain lobes. Merged images for histone H3 and mCD8GFP are also presented. (Scale bar, 50 μm.)

Supplementary Fig. 3. Characterization of *Baf* KD-induced defects in the formation of type I NB lineages

(a) kNN graphs embedded in Palantir t-SNE plots. The overall direction of arrows indicates developmental trajectories of type I NBs (type I NB – GMC – Neuron, *top*) and type II NBs (type II NB – INP – GMC – Neuron, *bottom*) in control (*left*) and *Baf* KD cells (*right*).

(b) Heatmap showing the Z-scores of average normalized expressions of 687 transcriptional regulators in control and *Baf* KD cells classified as type I NB lineage (*left*). Heatmap for Z-score of mean normalized expression of 692 transcriptional regulators grouped by each condition and cell types classified as type II NB lineage (*right*). Different colors at the axis between heatmaps are used to indicate group of genes sorted into 5 modules.

(c) GO enrichment analysis of upregulated (orange) and downregulated (magenta) genes in *Baf* KD cells classified as type I NB lineage.

(d) Heatmap showing the Z-scores of average normalized expressions of relevant genes classified in type I NB lineages (*left*) and II NB lineages (*right*).

(e) Representative Hoechst-stained and immunostained images for Mira (teal), aPKC (magenta) of cultured primary control type I NBs (*left*, *ase*-Gal4/+;UAS-*mCD8GFP*/+) and type I NBs expressing *Baf* RNAi (*right*, *ase*-Gal4/+;UAS-*mCD8GFP*/UAS-*Baf* RNAi) isolated from the brains of third-instar larvae. The white dashed lines in each panels indicate the outlines of NBs. (Scale bar, 10 μm.)

(f) Representative images of immunostaining for GABA (*top*), ChAT (*Middle*), and 5-HT (*Bottom*) in adult brains harboring following genotypes: *elav*-Gal4/+ and *elav*-Gal4/UAS-*Baf* RNAi. (Scale bar, 50 μm.)

(g) Quantification of the number of GABAergic, cholinergic, serotonergic neurons in brains of flies expressing the indicated transgenes (*elav*-Gal4/+ and *elav*-Gal4/UAS-*Baf* RNAi.). N.S., not significant according to Student's t-test; n=3 flies.

Supplementary Fig. 4. Characterization of behavioral abnormalities in adult flies lacking *Baf*

(a) Representative images showing wing postures of control flies (female, *upper left* and male, *lower left*) or flies expressing *Baf* RNAi driven by *elav*-Gal4 (female, *upper right* and male, *lower right*) (*elav*-Gal4/+ and *elav*-Gal4/UAS-*Baf* RNAi).

(b) Representative walking trajectory of a control fly (*elav*-Gal4/+) of *Baf* KD fly (*elav*-Gal4/UAS-*Baf* RNAi) in circular arena (Diameter: 1 cm) for 10 minutes.

(c) Aligned dot plots showing total travel distance of control flies (*elav*-Gal4/+) and *Baf* KD flies (*elav*-Gal4/UAS-*Baf* RNAi) for 10 minutes. **** $P < 1.0 \times 10^{-4}$ according to Student's t-test; error bars, mean ± SEM; the number of flies tested was as follows: Control = 10 flies, *Baf* KD = 7 flies.

(d) Aligned dot plots showing mean velocity of control flies (*elav*-Gal4/+) and *Baf* KD flies (*elav*-Gal4/UAS-*Baf* RNAi). **** $P < 1.0 \times 10^{-4}$ according to Student's t-test; error bars, mean ± SEM; the number of flies tested was as follows: Control = 10 flies, *Baf* KD = 7 flies.

(e) Box-and-whisker plots representing the 10th-90th percentiles with mean value (horizontal line) of total time spent on grooming and immobility (*Tub*-Gal80^{TS}/+;*elav*-Gal4/+ and *Tub*-Gal80^{TS}/+;*elav*-Gal4/UAS-*Baf* RNAi) for 10 minutes. Conditional expression of *Baf* RNAi in each experimental set (No KD, Adult KD, Developmental KD, or KD) is schematized above (E, embryo and A, adult). N.S., not significant; *** $P < 1.0 \times 10^{-3}$; **** $P < 1.0 \times 10^{-4}$ according to two-way ANOVA with Bonferroni post hoc test. n = 5 flies.

(f) Representative ethograms showing distinct behaviors of flies expressing the transgenes as follows (UAS-*Baf* RNAi/+, *nSyb*-Gal4/UAS-*Baf* RNAi, and *repo*-Gal4/UAS-*Baf* RNAi).

Different colors are used to distinguish different aspects of fly behaviors. n = 5.

(g) Box-and-whisker plots representing the 10th-90th percentiles with mean value (horizontal line) of total time spent on grooming and immobility in flies expressing the transgenes indicated in (*f*) for 10 minutes. N.S., not significant according to one-way ANOVA with Tukey's post hoc test; n = 5 flies.

Supplementary Fig. 5. Genetic screenings to identify downstream mediators of *Baf* KD-induced behavioral abnormalities

(a) GO terms of 19 genes that were downregulated (blue) genes inducing wing posture problems in adult flies

(b) Representative ethograms showing distinct behaviors of flies expressing the transgenes as follows (*ase*-Gal4/+, *ase*-Gal4/+;UAS-*Spindly* RNAi/+, *ase*-Gal4/+;UAS-*tsh* RNAi/+, *ase*-Gal4/+;UAS-*blw* RNAi/+, *ase*-Gal4/+;UAS-*msk* RNAi/+, *ase*-Gal4/UAS-*CG5033* RNAi, *ase*-Gal4/+;UAS-*Caf1-55* RNAi/+, *ase*-Gal4/+;UAS-*vvl* RNAi/+, *ase*-Gal4/+;UAS-*mts* RNAi, *ase*-Gal4/UAS-*Uba2* RNAi, *ase*-Gal4/UAS-*U4-U6-60K* RNAi, and *ase*-Gal4/+;UAS-*mars* RNAi/+). Different colors are used to distinguish different aspects of fly behavior. n = 5 flies.

(c) Box-and-whisker plots representing the 10th-90th percentiles with mean value (horizontal line) of total time spent on grooming and immobility in flies expressing following genotypes (*ase*-Gal4/+, *ase*-Gal4/+UAS-*tsh* RNAi/+, and *ase*-Gal4/+;UAS-*Spindly* RNAi/+) for 10 minutes. **** $P < 1.0 \times 10^{-4}$ according to one-way ANOVA with Tukey's post hoc test; n = 5 flies.

(d) Representative ethograms showing distinct behaviors of flies expressing the indicated transgenes (*ase*-Gal4/+;UAS-*Baf* RNAi/+, *ase*-Gal4/UAS-*Caf1-55*;UAS-*Baf* RNAi/+, and *ase*-Gal4/+;UAS-*Baf* RNAi/UAS-*mts*). Different colors are used to distinguish different aspects of fly behavior. n = 5 flies.

(e) Box-and-whisker plots representing the 10th-90th percentiles with mean value (horizontal line) of total time spent on grooming and immobility in flies expressing the transgenes indicated in (*d*) for 10 minutes. N.S., not significant according to one-way ANOVA with

Tukey’s post hoc test; n = 5 flies.

(f) Representative ethograms showing distinct behaviors of flies expressing the indicated transgenes (*ase*-Gal4/+, *ase*-Gal4/UAS-*Arm*, and *ase*-Gal4/+;UAS-*Arm* RNAi/+). n = 5 flies.

(g) Box-and-whisker plots representing the 10th-90th percentiles with mean value (horizontal line) of total time spent on grooming and immobility in flies expressing the transgenes indicated in (*f*) for 10 minutes. N.S., not significant; **** $P < 1.0 \times 10^{-4}$ according to one-way ANOVA with Tukey’s post hoc test; n = 5 flies.

Supplementary Fig. 6. Contribution of *tsh*, *Arm*, or *msk* to the formation of type I NBs and neural progenies

(a) Representative images of control type I NBs and type I NBs expressing *Baf* RNAi (*Baf* KD), *tsh* RNAi (*tsh* KD), and *Arm* RNAi (*Arm* KD) in the brains of third-instar larvae. (*ase*-Gal4/+;UAS-*mCD8GFP*,UAS-*H2A.mRFP*/+, *ase*-Gal4/+;UAS-*mCD8GFP*,UAS-*H2A.mRFP*/UAS-*Baf* RNAi, *ase*-Gal4/+;UAS-*mCD8GFP*,UAS-*H2A.mRFP*/UAS-*tsh* RNAi, and *ase*-Gal4/+;UAS-*mCD8GFP*,UAS-*H2A.mRFP*/UAS-*Arm* RNAi). The white dashed lines indicate the outlines of brain lobes. Magnified images of red squares in *top* panels presented in *bottom* panels. (Scale bar, 20 μm.)

(b) Quantification of the number of type I NBs located in the apical cortex of third-instar larval brain lobes expressing the transgenes indicated in (*a*). N.S., not significant, *** $P < 1.0 \times 10^{-3}$ according to one-way ANOVA with Tukey's post hoc test; the number of NBs tested was as follows: Control = 6 brain lobes, *Baf* KD = 4 brain lobes, *tsh* KD = 4 brain lobes, and *Arm* KD = 4 brain lobes.

(c) Quantification of the number of the neural progenies derived from type I NBs located in the apical cortex of third-instar larval brain lobes expressing the transgenes indicated in (*a*). N.S., not significant; **** $P < 1.0 \times 10^{-4}$ according to one-way ANOVA with Tukey's post hoc test; the number of brain lobes tested was as follows: Control = 15 NBs, *Baf* KD = 9 NBs, *tsh* KD = 12 NBs, and *Arm* KD = 9 NBs.

(d) Representative images of control type I NBs (*top*, *ase*-Gal4/+;UAS-*mCD8GFP*,UAS-*H2A.mRFP*/+) and type I NBs expressing *msk* RNAi (*bottom*, *ase*-Gal4/+;UAS-*mCD8GFP*,UAS-*H2A.mRFP*/UAS-*msk* RNAi) in the brains of third-instar larvae. The white dashed lines in the *left* panels show the outlines of brain lobes. Magnified images of red squares

in the *left* panels presented in the *right* panels. (Scale bars, 20 μm.)

(e) Quantification of the number of type I NBs in third-instar larval brain lobes expressing the transgenes indicated in (*d*). N.S., not significant according to Student's t-test; error bars, mean ± SEM; the number of brain lobes tested was as follows: Control = 9 brain lobes, *msk* KD = 4 brain lobes.

(f) Schematic diagrams for quantification of subnuclear positioning of heterochromatin. Confocal images of medial focal plane of NB were used for analyzing subnuclear position of heterochromatin. The nucleus of NBs divided into three equal area (central (3), intermediate (2), and peripheral (1) region of nucleus) of concentric rings.

(g) Representative images of immunostaining for Arm (magenta) in cultured primary type I NBs isolated from the brains of third-instar larvae expressing the transgenes as follows. (*ase*-Gal4/+; UAS-*mCD8GFP*/+). The white dashed lines in panels show the outlines of inner nuclear membrane. (Scale bar, 10 μm.)

(h) Representative images of immunostaining for Arm (magenta) in control type I NBs, type I expressing *Baf* RNAi (*Baf* KD), and *tsh* RNAi (*tsh* KD) in the brains of third-instar larvae. (*ase*-Gal4/+; UAS-*mCD8GFP*/+, *ase*-Gal4/+; UAS-*mCD8GFP*/UAS-*Baf* RNAi, *ase*-Gal4/+; UAS-*mCD8GFP*/UAS-*tsh* RNAi). (Scale bar, 10 μm.)

(i) Representative images of immunostaining for Baf (teal) and tsh (magenta) in cultured primary type I NBs isolated from the brains of third-instar larvae expressing the transgenes indicated as follows. (*ase*-Gal4/+;UAS-*mCD8GFP*/+, *ase*-Gal4/+, UAS-*mCD8GFP*/UAS-*Arm* RNAi). The white dashed lines in panels show the outlines of inner nuclear membrane. (Scale bar, 10 μm.)

(j) UMAP and violin plots showing key genes (*tsh* (*top* panel) and *Arm* (*bottom* panel)) in each experimental condition and cell type.

(k) Representative images of immunostaining for Baf (teal) and H3K9me3 (magenta) in cultured primary type I NBs isolated from the brains of third-instar larvae expressing the transgenes indicated as follows. (*ase*-Gal4/+;UAS-*mCD8GFP*/+, *ase*-Gal4/+, UAS-*mCD8GFP*/UAS-*tsh* RNAi). The white dashed lines in panels show the outlines of inner nuclear membrane. (Scale bar, 10 μm.)

Supplementary Fig. 7. Suppression of *Baf* KD-induced defects in the formation of type I NB lineages by overexpressing *tsh* or *Arm* in type I NBs.

(a) Scatter dot plots showing the proportion of mean intensity of heterochromatin localized in the peripheral zone of type I NB nucleus expressing the transgenes as follows. (*ase*-Gal4/+;UAS-*mCD8GFP*/+, *ase*-Gal4/+;UAS-*mCD8GFP*/UAS-*Baf* RNAi, *ase*-Gal4/UAS-*tsh*;UAS-*mCD8GFP*/UAS-*Baf* RNAi, *ase*-Gal4/UAS-*Arm*;UAS-*mCD8GFP*/UAS-*Baf* RNAi). * $P < 0.05$; **** $P < 1.0 \times 10^{-4}$ according to one-way ANOVA with Tukey's post hoc test; error bars, mean ± SEM; the number of type I NBs tested was as follows: Control = 12, *Baf* KD = 10, *tsh* KD = 14, and *Arm* KD = 9.

(b) Scatter dot plots showing the proportion of mean intensity of heterochromatin localized in the central zone of type I NB nucleus expressing the transgenes indicated in (*a*). *** $P < 1.0 \times 10^{-3}$; **** $P < 1.0 \times 10^{-4}$ according to one-way ANOVA with Tukey's post hoc test; error bars, mean ± SEM; the number of type I NBs tested was as follows: Control = 12, *Baf* KD = 10, *tsh* KD = 14, and *Arm* KD = 9.

(c) Representative images of immunostaining for Dpn (teal) and Pros (magenta) in control type I NBs, type I NBs expressing *Baf* RNAi (*Baf* KD), *Baf* RNAi + *tsh* (*Baf* KD + *tsh* OE), and *Baf* RNAi + *Arm* (*Baf* KD + *Arm* OE) in the brains of third-instar larvae. (*ase*-Gal4/+;UAS-*mCD8GFP*/+, *ase*-Gal4/+;UAS-*mCD8GFP*/UAS-*Baf* RNAi, *ase*-Gal4/UAS-*tsh*;UAS-*mCD8GFP*/UAS-*Baf* RNAi, *ase*-Gal4/UAS-*Arm*;UAS-*mCD8GFP*/UAS-*Baf* RNAi). The white dashed lines indicate the outlines of NBs (*second* row panels) and neural progenies (*third* row panels). (Scale bar, 20 µm.)

(d) Representative images of immunostaining for TH in the brains of adult flies expressing following the transgenes. (*ase*-Gal4/+, *ase*-Gal4/+;UAS-*Baf* RNAi/+, *ase*-Gal4/UAS-

tsh;UAS-*Baf* RNAi/+, *ase*-Gal4/UAS-*Arm*; UAS-*Baf* RNAi/+). (Scale bar, 50 µm.)

(e) Quantification of the number of dopaminergic neurons in the brains of flies expressing the transgenes indicated in (*d*). * $P < 0.05$; ** $P < 1.0 \times 10^{-2}$ by one-way ANOVA with Tukey's post hoc test; the number of brain lobes tested was as follows: Control = 3 brains, *Baf* KD = 3 brains, *Baf* KD + *tsh* OE = 3 brains, and *Baf* KD + *Arm* OE = 3 brains.

Supplementary tables with titles

Supplementary Table. 1. Sequence information for RT-PCR primers

**Gene**	**Sequences**
Baf	5'-TCGATATGGCCTACACCGTTTT<br>3'-ACGATTGGTGCGAGGAGTTC
RP49	5'-GCTTCAAGATGACCATCCGCCC<br>3'-GTTACGGATCGAACAAGCGCACC

Supplementary Table 2. Total number of analyzed cells

	**# cells with glia**	**# cells without glia**
Ctrl1	1637	1540
Ctrl2	1539	1459
**Ctrl**	**3176**	**2999**
KD1	1885	1745
KD2	1304	1205
**KD**	**3189**	**2950**
**Total**	**6365**	**5949**

Supplementary Table 3. List of differentially expressed genes between control and type I NB lacking *Baf*

Gene symbol	logFC	AveExpr	t	P.Value	adj.P.Val	B
Arc1	1.041116	0.663077	10.246	1.73E-19	6.40E-16	33.2009
Pzl	0.736311	0.4876289	7.804164	5.81E-13	7.18E-10	18.91907
CG45782	0.732692	0.5065254	7.838581	4.75E-13	7.04E-10	19.11016
CG17684	0.689587	0.5923901	7.260322	1.32E-11	1.09E-08	15.95259
DIP-lambda	0.667718	0.5063348	6.985982	6.12E-11	3.49E-08	14.49724
Myo81F	0.606021	1.0573794	5.932282	1.63E-08	4.64E-06	9.209301
Snap25	0.586616	0.4552308	6.486495	9.20E-10	3.79E-07	11.92705
CG14636	0.580329	0.3275125	7.309606	9.99E-12	9.25E-09	16.21709
OdsH	0.566778	0.2744238	8.075511	1.18E-13	2.18E-10	20.43565
CR10102	0.561026	0.2944939	7.559904	2.39E-12	2.53E-09	17.57403
Ac78C	0.539233	0.2692192	7.18317	2.04E-11	1.37E-08	15.54037
CG17574	0.53524	0.3095445	6.70979	2.78E-10	1.37E-07	13.06276
for	0.534598	0.7720869	5.499259	1.38E-07	3.29E-05	7.19561
Xrp1	0.522804	0.6039847	5.259424	4.30E-07	8.61E-05	6.125426
dikar	0.480652	0.8385552	4.647216	6.72E-06	0.0007654	3.552007
WDY	0.472012	0.4303935	5.131246	7.79E-07	0.0001443	5.567365
nrm	0.47142	0.2287635	6.856362	1.25E-10	6.61E-08	13.82005
rad50	0.458347	0.4036486	5.119097	8.24E-07	0.0001466	5.514988
CadN2	0.443989	0.4514645	4.879619	2.43E-06	0.0003602	4.501096
sima	0.424317	0.531115	4.587763	8.66E-06	0.0008912	3.314897
hpRNA:CR46342	0.423913	0.4426439	4.680374	5.82E-06	0.0007005	3.685267
Ir40a	0.422719	0.2686481	5.869083	2.24E-08	6.14E-06	8.909135
CG32767	0.416309	0.4749634	4.383824	2.04E-05	0.0016579	2.519629
dpy	0.407664	0.1868966	6.31462	2.28E-09	8.44E-07	11.06833
CG3726	0.397225	0.271082	5.392843	2.29E-07	5.14E-05	6.716651
hang	0.39498	0.762508	3.913933	0.000131	0.0073048	0.798067
ImpL2	0.39483	0.1907258	6.102837	6.84E-09	2.20E-06	10.02959
vn	0.393793	0.1845125	6.344628	1.95E-09	7.59E-07	11.21725
scyl	0.391354	0.4581076	4.32079	2.64E-05	0.0020145	2.279592
Dbp80	0.388255	0.9554366	3.797021	0.000204	0.0103249	0.394697
tai	0.3836	0.6141029	3.977575	0.000103	0.0062956	1.021907
Nipped-B	0.38355	0.7297007	4.03845	8.13E-05	0.005235	1.238795
CG43861	0.378637	0.1987135	5.938698	1.58E-08	4.64E-06	9.239887
MRP	0.376262	0.3540386	4.305719	2.81E-05	0.0021203	2.222612

CG14073	0.373234	0.3769702	4.463228	1.46E-05	0.0012917	2.825905
CG40006	0.372229	0.2867995	4.628011	7.29E-06	0.0008064	3.475156
Lasp	0.367482	0.5229517	3.992651	9.70E-05	0.0060917	1.075367
CG6686	0.359752	0.32528	4.428261	1.69E-05	0.0014666	2.690501
Ppr-Y	0.359207	0.2434768	4.636635	7.03E-06	0.0007889	3.509638
MFS17	0.353415	0.4508483	3.669582	0.000325	0.0147611	-0.03327
toc	0.352909	0.6801289	3.606873	0.000407	0.0173053	-0.23931
kl-3	0.349177	0.2154354	5.195419	5.79E-07	0.0001129	5.845528
lncRNA:Hsromega	0.348775	1.360903	3.549326	0.0005	0.0201082	-0.42572
AlaRS	0.344211	0.2963874	4.377184	2.09E-05	0.0016854	2.494215
Taf1	0.341744	0.2614112	4.759626	4.13E-06	0.000566	4.006694
poe	0.338743	0.8253978	3.282749	0.001248	0.0375698	-1.25537
tud	0.337021	0.509837	3.474294	0.00065	0.0247227	-0.6649
unc-13	0.331824	0.738596	3.221429	0.001529	0.0438841	-1.4382
foi	0.330286	0.2804317	4.141783	5.42E-05	0.0037164	1.613114
Xpc	0.319128	0.6251412	3.207899	0.001598	0.0448339	-1.47813
AGO3	0.317804	0.3443171	3.740174	0.000251	0.0119203	0.202271
Dgp-1	0.317635	0.1913204	4.815295	3.23E-06	0.0004624	4.234914
gpp	0.314208	0.5976487	3.211026	0.001582	0.0445454	-1.46892
shep	0.312697	0.4704114	3.370791	0.000928	0.0302698	-0.98759
Neto	0.310216	0.2379315	4.510945	1.20E-05	0.0011536	3.012029
CG42795	0.308718	0.1697008	4.97402	1.59E-06	0.0002566	4.8965
Smurf	0.308583	0.3830985	3.461427	0.00068	0.0252926	-0.70548
l(3)psg2	0.306769	0.4434929	3.244131	0.001418	0.0410389	-1.37086
Gprk2	0.306434	0.4037725	3.473659	0.000651	0.0247227	-0.66691
CG34347	0.305839	0.1913286	4.678752	5.86E-06	0.0007005	3.678732
CG33158	0.305365	0.5006567	3.245868	0.00141	0.040964	-1.36569
Dg	0.304084	0.3410332	3.618192	0.000391	0.0171336	-0.20235
CG30389	0.303827	0.3280205	3.924142	0.000126	0.0070799	0.83377
RhoGAP93B	0.299685	0.2774661	3.719056	0.000271	0.0126382	0.131409
kek1	0.29434	0.1410786	5.318038	3.27E-07	6.72E-05	6.38388
Ect4	0.294268	0.2149857	4.236378	3.71E-05	0.0027508	1.96249
nvd	0.293362	0.1959228	4.505784	1.23E-05	0.0011638	2.991826
CG32369	0.293242	0.149879	4.91286	2.10E-06	0.0003169	4.639686
mld	0.292427	0.3513244	3.439602	0.000733	0.026259	-0.774
CG7956	0.28955	0.1737898	4.589082	8.61E-06	0.0008912	3.32013
CG7457	0.286604	0.1733037	4.696552	5.43E-06	0.0006933	3.750544
CG15784	0.28545	0.1343031	4.748521	4.33E-06	0.0005757	3.961408

pcs	0.285444	0.3277588	3.437143	0.000739	0.026259	-0.7817
Pvf2	0.284249	0.1462071	4.988211	1.49E-06	0.000246	4.956424
CG4022	0.283585	0.2010921	4.193476	4.41E-05	0.003169	1.803248
bru1	0.278707	0.2013973	3.764898	0.000229	0.0111703	0.285661
Ldh	0.276892	0.1415501	4.423988	1.72E-05	0.0014686	2.674011
hig	0.275597	0.3269524	3.408783	0.000815	0.0273495	-0.87012
Usp2	0.273075	0.3423845	3.312113	0.001131	0.0350208	-1.16674
CG8963	0.272744	0.295848	3.453868	0.000698	0.0257342	-0.72925
dnc	0.272245	0.3542136	3.180607	0.001747	0.047572	-1.55823
CG6701	0.270314	0.2643795	3.479867	0.000637	0.0245875	-0.64729
Teh1	0.269836	0.1597093	4.501015	1.25E-05	0.0011724	2.973172
Tep4	0.267596	0.1221422	5.11707	8.31E-07	0.0001466	5.506258
osp	0.266081	0.2221368	3.826411	0.000182	0.0095821	0.495138
RtcB	0.2615	0.1302553	4.93047	1.94E-06	0.000299	4.713389
CG3408	0.252875	0.194401	3.525246	0.000544	0.021498	-0.50296
Ptpmeg2	0.250729	0.1520283	4.019517	8.75E-05	0.0055856	1.17105
kirre	0.249192	0.2589658	3.476914	0.000644	0.0247128	-0.65662
CG43658	0.248975	0.2340806	3.399986	0.00084	0.0280175	-0.89742
dpr6	0.248224	0.1409215	4.41181	1.81E-05	0.0015271	2.627084
stc	0.247486	0.2075211	3.738461	0.000253	0.0119203	0.196511
CadN	0.246218	0.1959526	3.524263	0.000546	0.021498	-0.5061
hpRNA:CR32207	0.244902	0.1277735	4.476	1.39E-05	0.0012544	2.875574
trr	0.243663	0.1700238	3.691913	0.0003	0.0136955	0.04083
CG10492	0.239039	0.2238097	3.249455	0.001394	0.0406404	-1.35501
FoxK	0.232041	0.2251462	3.260888	0.001342	0.0394406	-1.32089
MBD-R2	0.228015	0.1936255	3.374102	0.000917	0.0300631	-0.97739
cv-c	0.227793	0.1880534	3.432113	0.000752	0.0264095	-0.79743
cher	0.227001	0.1887816	3.490329	0.000615	0.0239564	-0.61416
melt	0.225545	0.1026882	4.577708	9.04E-06	0.000905	3.275027
Gadd45	0.225117	0.1050482	4.283085	3.08E-05	0.0023011	2.137332
brun	0.222379	0.1401571	3.65538	0.000342	0.0153509	-0.0802
nAChRalpha4	0.220773	0.191429	3.335907	0.001044	0.0329065	-1.09443
Corp	0.21617	0.1137028	4.081504	6.87E-05	0.0045037	1.393822
scro	0.215318	0.1364651	3.666435	0.000329	0.0148392	-0.04368
Imp	0.212366	0.1553459	3.388384	0.000874	0.0287621	-0.93333
DNApol-zeta	0.21075	0.1699389	3.349807	0.000996	0.032079	-1.05197
dpr21	0.21072	0.1258334	3.785294	0.000213	0.0105392	0.354803
lawc	0.210634	0.1352624	3.964204	0.000108	0.0064636	0.974633

Ugt50B3	0.209068	0.098985	3.951842	0.000114	0.0065885	0.931038
EloA	0.20823	0.1924381	3.179069	0.001756	0.0476355	-1.56272
CG6424	0.206906	0.1345326	3.396199	0.000851	0.0282555	-0.90916
CG10631	0.203976	0.1664822	3.339235	0.001032	0.0327868	-1.08427
rdog	0.203956	0.0919259	4.469252	1.43E-05	0.0012748	2.849316
CG41520	0.203326	0.1109726	4.065943	7.30E-05	0.0047444	1.337633
Axud1	0.203309	0.1411148	3.606232	0.000408	0.0173053	-0.2414
REPTOR	0.203307	0.1899956	3.181689	0.001741	0.047572	-1.55507
Apc	0.202981	0.1216351	3.605662	0.000409	0.0173053	-0.24326
CG30069	0.194351	0.1221487	3.61316	0.000398	0.0172932	-0.21879
CycY	0.193192	0.1451229	3.309901	0.001139	0.0350208	-1.17344
LanA	0.193161	0.1013256	4.086133	6.75E-05	0.0044622	1.410567
l(2)01289	0.190651	0.0874969	3.962243	0.000109	0.0064636	0.967709
CG6664	0.189803	0.1247039	3.601057	0.000416	0.0173588	-0.25827
CG34357	0.187668	0.1013298	3.582396	0.000444	0.0182805	-0.31891
mbc	0.185547	0.1363065	3.198399	0.001648	0.0458118	-1.50608
CG12163	0.183235	0.1431707	3.264889	0.001324	0.0390755	-1.30893
sca	0.182817	0.084337	3.954512	0.000112	0.0065885	0.940446
dpr8	0.182353	0.0835851	4.324343	2.60E-05	0.0020062	2.293049
ds	0.178286	0.0783942	4.087865	6.70E-05	0.0044622	1.416839
Marf1	0.178163	0.096911	3.624862	0.000382	0.016863	-0.18051
Piezo	0.172667	0.1263824	3.266729	0.001316	0.0389928	-1.30342
sls	0.171131	0.1035792	3.471824	0.000655	0.0247227	-0.6727
Tes	0.170426	0.1033667	3.338572	0.001035	0.0327868	-1.0863
Strn-Mlck	0.161646	0.0902467	3.310723	0.001136	0.0350208	-1.17096
Mocs1	0.1611	0.0744434	3.84082	0.000173	0.009144	0.544616
CG9003	0.159073	0.0842465	3.424758	0.000771	0.0266962	-0.82039
RhoGEF64C	0.148803	0.0706237	3.324014	0.001087	0.0339628	-1.13063
fj	0.148608	0.079347	3.449267	0.000709	0.0257342	-0.7437
cwo	0.147866	0.065309	3.791119	0.000208	0.0104818	0.374607
Mkp3	0.147218	0.0793364	3.412064	0.000806	0.0272498	-0.85993
CG33978	0.147178	0.0658843	3.811401	0.000193	0.0099229	0.443762
CG34383	0.146362	0.0825781	3.187535	0.001708	0.0470289	-1.53795
Oatp30B	0.14353	0.0906744	3.237351	0.00145	0.0418047	-1.39102
qin	0.141173	0.091238	3.173105	0.00179	0.0483913	-1.58014
LanB1	0.140917	0.0652349	3.545039	0.000507	0.0203047	-0.43951
Shrm	0.139337	0.065474	3.560979	0.000479	0.0195081	-0.38818
Drip	0.131135	0.0609919	3.491852	0.000611	0.0239555	-0.60933

CG7900	0.128836	0.0693908	3.192957	0.001678	0.0463767	-1.52206
Ser	0.118511	0.0547473	3.453545	0.000698	0.0257342	-0.73027
IA-2	0.116148	0.0517296	3.357123	0.000972	0.0314305	-1.02956
CG14304	0.110028	0.0499254	3.334396	0.001049	0.0329345	-1.09903
hid	0.10674	0.0489921	3.167965	0.00182	0.0490269	-1.59513
Mer	0.101432	0.045323	3.273232	0.001288	0.0384668	-1.28394
Tbc1d15-17	-0.1452	0.0903518	-3.18379	0.001729	0.0474305	-1.54892
foxo	-0.14626	0.0803743	-3.43199	0.000752	0.0264095	-0.7978
tsh	-0.15083	0.0833082	-3.31323	0.001127	0.0350208	-1.16335
Gbs-70E	-0.16192	0.098394	-3.41783	0.00079	0.027087	-0.842
ZnT49B	-0.16538	0.091464	-3.46928	0.000661	0.0247369	-0.68074
E(spl)mgamma-HLH	-0.16646	0.0922957	-3.58259	0.000444	0.0182805	-0.31829
ATPsynO	-0.16892	0.0927769	-3.60784	0.000406	0.0173053	-0.23617
CG13305	-0.17754	0.109134	-3.40835	0.000816	0.0273495	-0.87147
CG12288	-0.1824	0.0999949	-3.81587	0.00019	0.0098963	0.459049
CG44153	-0.19636	0.1519937	-3.34033	0.001029	0.0327868	-1.08095
CG13551	-0.19779	0.1383383	-3.44931	0.000709	0.0257342	-0.74356
CG5033	-0.20057	0.1766686	-3.20611	0.001607	0.0449274	-1.4834
Idh3b	-0.20923	0.1274841	-3.73115	0.00026	0.0121669	0.171962
Spindly	-0.21128	0.1406762	-3.76281	0.000231	0.011183	0.27861
CG1607	-0.23132	0.2255128	-3.26773	0.001312	0.0389928	-1.30043
lncRNA:CR44024	-0.23609	0.249244	-3.29149	0.001212	0.0370928	-1.22905
CCT1	-0.24349	0.234084	-3.27341	0.001287	0.0384668	-1.28341
HIP	-0.24871	0.3029499	-3.21103	0.001582	0.0445454	-1.46889
Aos1	-0.24998	0.2242485	-3.56288	0.000476	0.0194842	-0.38205
vvl	-0.2589	0.1901474	-4.11123	6.11E-05	0.0041167	1.501642
mars	-0.26616	0.2525045	-3.75573	0.000237	0.0114044	0.254691
RpS15	-0.26757	0.2890735	-3.38881	0.000872	0.0287621	-0.93203
Eph	-0.27786	0.322244	-3.4709	0.000658	0.0247227	-0.67562
His-Psi:CR31754	-0.28349	0.1914201	-4.38533	2.02E-05	0.0016579	2.525382
RpLP2	-0.28462	0.199546	-4.39744	1.93E-05	0.0016025	2.57183
Pex7	-0.28972	0.2977464	-3.60118	0.000415	0.0173588	-0.25787
RpS24	-0.29065	0.3330828	-3.60004	0.000417	0.0173588	-0.26157
feo	-0.29121	0.5851454	-3.22022	0.001535	0.0438885	-1.44177
mt:ND2	-0.29179	0.39879	-3.43641	0.000741	0.026259	-0.78398
CG12496	-0.29256	0.2080279	-4.19134	4.45E-05	0.003169	1.79535
Rap1	-0.29332	0.4274549	-3.41862	0.000788	0.027087	-0.83951
Ote	-0.29778	0.1926923	-4.74742	4.35E-06	0.0005757	3.95692

U4-U6-60K	-0.29813	0.3116083	-3.86089	0.00016	0.0086659	0.61381
insc	-0.29899	0.4532429	-3.21886	0.001542	0.0439153	-1.44578
His2B:CG33908	-0.29935	1.1011339	-3.28729	0.001229	0.0371691	-1.24172
Cadps	-0.30416	0.4268371	-3.2533	0.001376	0.0402848	-1.34354
His2B:CG33876	-0.30489	0.2293893	-4.66606	6.19E-06	0.0007282	3.62766
Hsc70Cb	-0.30845	0.9140882	-3.21108	0.001581	0.0445454	-1.46874
RpL24	-0.31149	0.3312625	-3.88226	0.000148	0.0080543	0.687773
Hsp83	-0.31307	1.4803769	-3.35994	0.000962	0.0312684	-1.02093
Caf1-55	-0.31307	0.5366637	-3.33825	0.001036	0.0327868	-1.08728
lncRNA:CR30009	-0.31374	0.2829899	-3.88545	0.000146	0.0080489	0.698872
His1:CG33855	-0.31567	0.2581302	-4.42711	1.70E-05	0.0014666	2.68604
puf	-0.31623	0.4818592	-3.41273	0.000804	0.0272498	-0.85785
Hsp70Bc	-0.31678	0.4238224	-3.44704	0.000714	0.0258066	-0.75069
Rrp1	-0.31827	0.5455912	-3.42803	0.000763	0.026521	-0.81018
Task6	-0.31941	0.3465947	-3.93726	0.00012	0.0068095	0.879748
mts	-0.31988	0.7357695	-3.28719	0.001229	0.0371691	-1.24202
His2A:CG33808	-0.32027	0.4449967	-3.43058	0.000756	0.0264135	-0.80223
CG1910	-0.32419	0.6100031	-3.41526	0.000797	0.0272007	-0.84998
His1:CG33813	-0.32438	0.4041351	-3.64643	0.000353	0.0157592	-0.10968
RpL41	-0.32495	0.3014413	-4.20721	4.18E-05	0.0030324	1.854089
mt:ND1	-0.32549	0.4068445	-3.88439	0.000147	0.0080489	0.695171
His2B:CG33872	-0.32587	0.2633205	-4.71572	5.00E-06	0.0006492	3.828094
polo	-0.32805	0.4672125	-3.70996	0.00028	0.0129839	0.100983
CG7839	-0.32864	0.3602981	-3.85837	0.000162	0.0086853	0.605085
eIF4A	-0.3301	0.8634016	-3.19785	0.001651	0.0458118	-1.50769
Hsp68	-0.33154	0.5259989	-3.28747	0.001228	0.0371691	-1.24115
Vap33	-0.3316	0.763605	-3.45045	0.000706	0.0257342	-0.73999
gukh	-0.33252	0.5769044	-3.43807	0.000737	0.026259	-0.7788
SoxN	-0.33438	0.4487268	-3.74595	0.000246	0.0117463	0.221711
smid	-0.33506	0.3980945	-3.93635	0.00012	0.0068095	0.876579
mt:CoI	-0.33748	1.1736589	-3.70121	0.00029	0.0133216	0.071805
B52	-0.33784	0.5223341	-3.48853	0.000618	0.0239812	-0.61987
Ranbp9	-0.33871	0.3562085	-4.01147	9.02E-05	0.005713	1.142351
vig	-0.34488	0.4644061	-3.94141	0.000118	0.006783	0.894327
blw	-0.34529	0.9977388	-3.5549	0.00049	0.0198237	-0.40779
smt3	-0.34716	0.7174098	-3.7842	0.000213	0.0105392	0.351087
His2B:CG33868	-0.35136	1.3849812	-3.98928	9.83E-05	0.0061041	1.063413
CG43340	-0.35454	0.3188953	-4.49592	1.28E-05	0.0011828	2.953257

Ald1	-0.35455	0.5237666	-3.80246	0.000199	0.0101881	0.413225
Hsp70Ba	-0.35839	0.4979658	-3.53462	0.000526	0.0209511	-0.47293
HmgZ	-0.35956	0.3689538	-4.32593	2.58E-05	0.0020062	2.299062
Mapmodulin	-0.36271	0.8490091	-3.95105	0.000114	0.0065885	0.928266
Dsp1	-0.36469	0.7083815	-3.78923	0.000209	0.0104843	0.368175
hth	-0.36624	0.5639186	-3.81265	0.000192	0.0099229	0.448026
msk	-0.36714	0.6855566	-3.61232	0.000399	0.0172932	-0.22153
His1:CG33834	-0.37085	0.5609969	-3.97401	0.000104	0.0063301	1.009293
Top2	-0.37268	1.1207684	-3.76882	0.000226	0.0110827	0.298919
His1:CG33801	-0.37562	0.7918096	-3.62428	0.000382	0.016863	-0.1824
alphaTub84B	-0.37834	1.2375877	-3.98776	9.89E-05	0.0061041	1.057999
Cp1	-0.3787	0.4059364	-4.35777	2.27E-05	0.0018059	2.420098
clu	-0.38232	0.412357	-4.35514	2.29E-05	0.0018061	2.410059
His3:CG33818	-0.38259	0.3962217	-4.65882	6.39E-06	0.0007395	3.59856
CG43736	-0.3852	0.4039723	-4.61314	7.77E-06	0.0008345	3.415812
msps	-0.38537	0.627974	-4.12385	5.82E-05	0.0039528	1.547598
Rcc1	-0.38927	0.7043754	-3.9689	0.000106	0.0064031	0.991221
CycA	-0.38974	0.813269	-3.8528	0.000165	0.0088052	0.585861
His1:CG33861	-0.39804	0.6120937	-4.1609	5.02E-05	0.00351	1.683214
HmgD	-0.40371	1.137073	-4.14731	5.30E-05	0.0036699	1.633355
mt:ATPase8	-0.4061	0.6091865	-4.16389	4.96E-05	0.0035016	1.694187
Vha16-1	-0.41027	0.5808026	-4.48809	1.32E-05	0.0012072	2.922694
Drak	-0.41311	0.5338338	-4.59486	8.40E-06	0.0008893	3.343087
Pen	-0.42848	1.1651546	-4.58047	8.94E-06	0.000905	3.285959
Uba2	-0.43215	0.5569248	-4.67972	5.84E-06	0.0007005	3.682649
lncRNA:CR31386	-0.44265	0.5568435	-4.8143	3.25E-06	0.0004624	4.23081
E2f1	-0.44434	0.8010706	-4.61914	7.58E-06	0.0008253	3.439743
grh	-0.44592	0.7870778	-4.53904	1.07E-05	0.0010382	3.122321
mod	-0.44739	0.903463	-4.54235	1.05E-05	0.0010374	3.135374
CG46301	-0.44777	0.8686022	-4.23395	3.75E-05	0.0027508	1.953433
nab	-0.47202	0.3787992	-5.82277	2.82E-08	7.21E-06	8.690506
ATPsynC	-0.47517	0.9198717	-4.68266	5.77E-06	0.0007005	3.694472
betaTub56D	-0.48007	1.1026094	-4.96862	1.63E-06	0.0002573	4.873711
mt:CoII	-0.48263	0.6069935	-5.40287	2.19E-07	5.06E-05	6.761509
mt:ATPase6	-0.49403	0.7467484	-5.02647	1.26E-06	0.0002166	5.118582
CG15646	-0.49822	0.5158781	-5.34114	2.93E-07	6.20E-05	6.48629
His2A:CG33865	-0.51473	0.7627999	-4.79194	3.58E-06	0.0005006	4.138927
His1:CG33816	-0.52307	0.8879154	-5.18908	5.96E-07	0.0001133	5.817953

jigr1	-0.52822	0.7215955	-5.35297	2.77E-07	6.03E-05	6.538884
lncRNA:CR33938	-0.53363	0.8491163	-4.99101	1.48E-06	0.000246	4.968241
His3:CG33821	-0.53607	0.4032359	-6.65028	3.83E-10	1.77E-07	12.75793
lncRNA:CR45242	-0.55427	0.3469616	-7.07529	3.73E-11	2.30E-08	14.96781
mt:Cyt-b	-0.55908	0.9151719	-5.72186	4.66E-08	1.15E-05	8.218076
CG6695	-0.55959	0.4881922	-6.24289	3.31E-09	1.12E-06	10.71407
CycB3	-0.56216	0.7267825	-6.01779	1.06E-08	3.26E-06	9.618702
Hs6st	-0.5747	1.1570504	-5.85316	2.42E-08	6.41E-06	8.833827
CycE	-0.58574	0.7176344	-6.29623	2.51E-09	8.85E-07	10.97725
Syp	-0.61875	1.5524054	-6.52506	7.49E-10	3.27E-07	12.1216
corto	-0.63454	0.4263165	-7.20253	1.83E-11	1.35E-08	15.64359
stg	-0.79077	0.6959596	-8.29822	3.13E-14	7.72E-11	21.69667
Eip93F	-0.89959	0.581011	-10.6879	1.01E-20	7.49E-17	35.89342

Supplementary information accompanies the manuscript on the Experimental & Molecular Medicine website (http://www.nature.com/emm/)
